# Combined FXIII-C3 autoantibodies elicit bleeding and complement dysfunction in autoimmune FXIII deficiency

**DOI:** 10.1172/JCI192619

**Published:** 2025-12-09

**Authors:** Shanshan Luo, Jun Deng, Yue Liu, Lv Xiong, Wanting Wang, Chaofan Wang, Yaohua Cai, Yajie Ding, Bahgat Fayed, Zhipeng Cheng, Lu Zhang, Min Zhang, Jun Fang, Gensheng Zhang, Rui Zhu, Haiqiang Jiang, Yunlun Li, Kun Huang, Xiang Cheng, Liang V. Tang, Chunyan Sun, Heng Mei, Peter F. Zipfel, Huafang Wang, Yadan Wang, Desheng Hu, Yu Hu

**Affiliations:** 1 Institute of Hematology, Union Hospital, Tongji Medical College, Huazhong University of Science and Technology, Wuhan, China.; 2 National Clinical Research Center for Chinese Medicine Cardiology, Xiyuan Hospital, Chinese Academy of Chinese Medical Sciences, Beijing, China.; 3 Department of Chemistry of Natural and Microbial Products, Pharmaceutical and Drug Industries Research Institute, National Research Centre, Giza, Egypt.; 4 Department of Critical Care Medicine, Second Affiliated Hospital, Zhejiang University School of Medicine, Hangzhou, Zhejiang, China.; 5 Department of Integrated Traditional Chinese and Western Medicine, Union Hospital, Tongji Medical College, Huazhong University of Science and Technology, Wuhan, China.; 6 Innovation Research Institute of Traditional Chinese Medicine, Shandong University of Traditional Chinese Medicine, Jinan, China.; 7 School of Pharmacy,; 8 Department of Cardiology, Union Hospital, and; 9 Hubei Key Laboratory of Biological Targeted Therapy, Union Hospital, Tongji Medical College, Huazhong University of Science and Technology, Wuhan, China.; 10 Department of Infection Biology, Leibniz Institute for Natural Product Research and Infection Biology, Hans Knöll Institute, Jena, Germany.; 11 Friedrich Schiller University, Faculty of Biological Sciences, Jena, Germany.; 12 Key Lab of Molecular Biological Targeted Therapies of the Ministry of Education, Union Hospital, Tongji Medical College, Huazhong University of Science and Technology, Wuhan, China.

**Keywords:** Autoimmunity, Infectious disease, Autoimmune diseases, Coagulation, Complement

## Abstract

Autoimmune factor XIII (FXIII) deficiency is a rare hemorrhagic disease characterized by severe bleeding and a high mortality rate. However, the pathogenesis of this disease remains unclear. FXIII consumption caused by infections is becoming increasingly common. Our clinical investigation, combined with in vivo experiments, revealed that patients and mice with autoimmune FXIII deficiency displayed complement dysfunction and that pathogenic infection and autoantibody generation were positively correlated. Further analysis revealed the presence of combined FXIII-C3 autoantibodies in patients with autoimmune FXIII deficiency. These combined autoantibodies neutralize FXIII, cause bleeding, and form a complex with C3, inhibiting complement activation and complement-mediated adaptive immune responses. Therefore, compromised immune responses increase host susceptibility to pathogenic *Candida albicans* infections. Consequently, uncontrolled exogenous fungal infections further activate platelets and cause platelet-related CD40 ligand (CD40L) release. By interacting with the CD40 on the B cell surface, the released CD40L further promotes autoreactive B cell activation to produce more autoantibodies, thereby forming a self-amplification loop for the progressive consumption of FXIII. We believe this study provides a perspective on disease pathogenesis and therapeutic guidance for better treatment of autoimmune FXIII deficiency.

## Abstract 2

<strong>Disease Associated Combined FXIII-C3</strong> <strong>Autoantibodies Trigger a Self-</strong><strong>A</strong><strong>mplification Loop for Progressive Depletion of FXIII </strong><strong>*via*</strong> <strong>Complement Dysfunction and </strong><strong>P</strong><strong>latelet</strong><strong>-</strong><strong>released CD154</strong>

## Graphical abstract

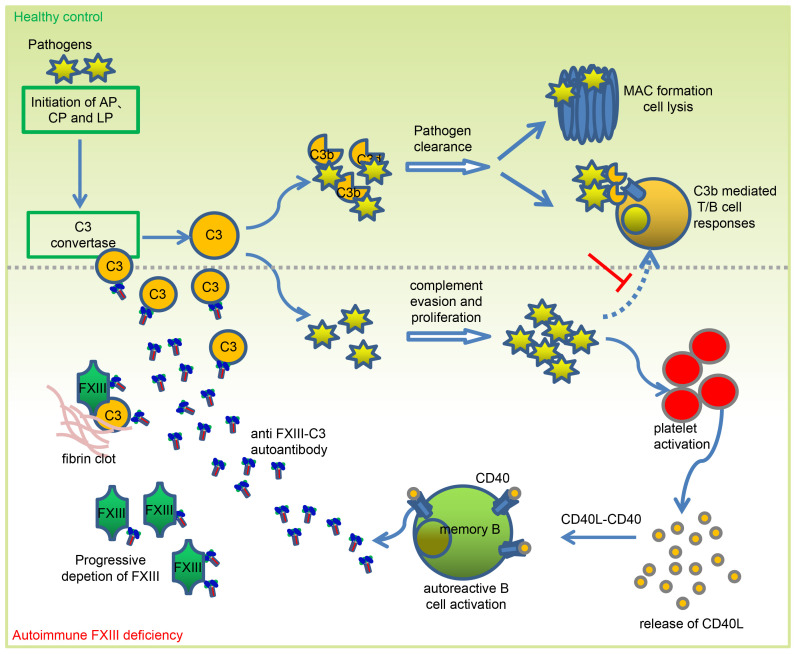

## Introduction

Autoimmune factor XIII (FXIII), a key molecule at the intersection of coagulation and fibrinolysis, is critical for promoting coagulation and hemostasis. In the absence of FXIII, the body may experience recurrent spontaneous bleeding with a wide range of bleeding sites (1). Clinically diagnosed FXIII deficiency can be classified as congenital or acquired deficiency. Acquired FXIII deficiency can be further divided into autoimmune and nonimmune-related deficiencies. Autoimmune FXIII deficiency is characterized by defective FXIII levels, severe hemorrhagic diathesis, and autoantibodies against FXIII, but the disease pathogenesis is unknown. Nonimmune-related deficiencies are characterized by defective FXIII levels, but no autoantibodies are developed, and the deficiencies are normally caused by acute consumption of FXIII because of various primary diseases, including leukemia, myelodysplastic syndrome, liver disease, disseminated intravascular coagulation (DIC), among others (2, 3).

Autoimmune FXIII deficiency is a rare hemorrhagic disease characterized by severe bleeding and high mortality rates (4). However, recent reports have found that the incidence of autoimmune FXIII deficiency is increasing annually, with a high number of cases reported in Japan (5). The currently available clinical treatment for patients with autoimmune FXIII deficiency involves the administration of immunosuppressants to remove autoantibodies, along with FXIII alternative therapy to stop bleeding. However, the treatment efficacy remains suboptimal. Therefore, a deep exploration of disease pathogenesis is urgently needed to help develop specific immunotherapies for clinical intervention and improve the cure rate of patients.

Among the numerous environmental factors, infection is 1 of the most substantial contributors to the development of autoimmune diseases (6). The quantity of autoantibody production is closely related to the susceptibility of the host (7, 8). This process is accompanied by platelet activation (9) and platelet-related CD40 ligand (CD40L) release, which is believed to be critical for triggering autoreactive B cell activation (10). When patients already have an autoimmune deficiency, autoimmune signals are amplified upon infection (11). Although Ichinose et al. (12) reported that approximately half of the autoimmune FXIII deficiency cases were idiopathic, followed by autoimmune diseases and solid tumors, FXIII consumption caused by infection is becoming increasingly common in clinical settings. As reported, 74% of patients in an intensive care unit (ICU) who had COVID-19 had lower FXIII antigen levels and activity than did noninfected individuals, and FXIII was continuously consumed during hospitalization (13). These clinical reports suggest infection is likely a key factor that can trigger autoimmune FXIII deficiency. However, the underlying molecular mechanism of infection-related autoimmune FXIII deficiency remains unclear.

In this study, we aimed to reveal whether and how pathogenic infections are involved in the progression of autoimmune FXIII deficiency by investigating patients with different types of FXIII deficiency, applying in vivo mouse models, and conducting in vitro molecular analysis. We believe this study will provide a perspective on disease pathogenesis and therapeutic guidance for better treatment of autoimmune FXIII deficiency.

## Results

### Patients with autoimmune FXIII deficiency had decreased complement activation.

**Figure 1:**
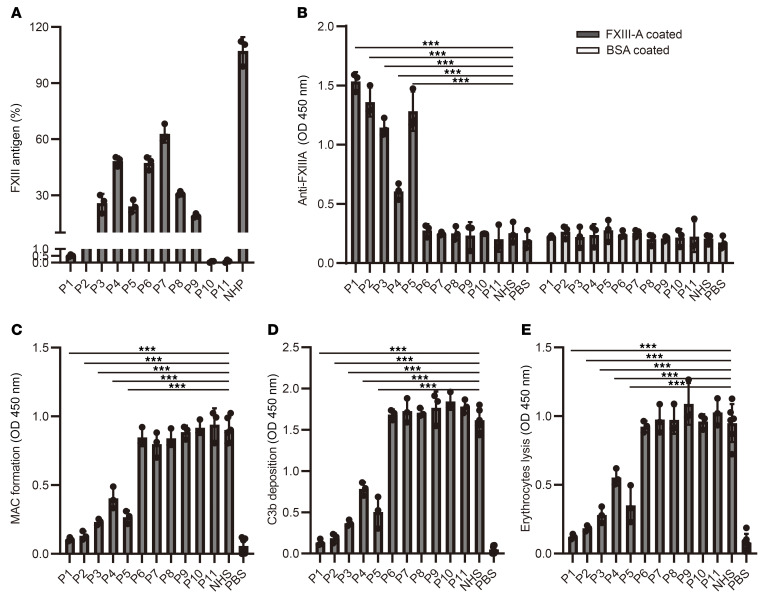
Patients with autoimmune FXIII deficiency had decreased complement activation. (**A**) FXIII antigen level. (**B**) Autoantibodies against FXIII-A detected in patients with different types of FXIII deficiency. (**C** and **D**) Complement activation of AP in all patients as well as healthy control individuals as indicated by MAC formation (**C**) and C3b/iC3b deposition (**D**). (**E**) Hemolytic assay. In this assay, different patients’ serum and NHS were incubated with rabbit erythrocytes, then erythrocyte lysis was recorded to quantify complement activation. Data are shown as mean ± SD. One-way ANOVA was used for statistical analysis. ***P < 0.001.

Although autoimmune FXIII deficiency is a rare hemorrhagic disease, it is associated with severe bleeding and a high mortality rate (14). In the past 5 years, we identified patients with 3 different types of FXIII deficiencies (see the flow chart depicted in Supplemental Figure 1A; supplemental material available online with this article; https://doi.org/10.1172/JCI192619DS1). Five patients with reduced FXIII-A antigen levels (0.5%, 4.8%, 26.9%, 49.1%, and 27.6%, respectively) and detectable autoantibodies in plasma were classified as patients with immune deficiency (patients 1–5 [P1–P5]). Four patients with decreased FXIII-A antigen levels (48.3%, 68.2%, 30.5%, and 18.1%, respectively), but without detectable autoantibodies, were classified as being nonimmune-deficient (P6–P9). Two additional patients with genetically confirmed FXIII deficiency were classified as having congenital deficiency (P10 and P11) (Table 1 and Figure 1, A and B). All these patients had coagulation disorders but normal prothrombin time, activated partial thromboplastin time, thrombin time, and FXIII-B antigen levels (Table 1 and Supplemental Table 1). Bleeding scores of all patients listed in Table 1 were evaluated based on the WHO bleeding grading system according to the patients’ first medical record. Meanwhile, 5 healthy donors were included as control participants. Autoantibodies developed in P1–P5 were specific to FXIII-A but not FXIII-B (Figure 1B and Supplemental Figure 1B).

Among the 5 patients with autoimmune deficiency, P1, P3, and P5 had an infection when diagnosed. Further screening of pathogen sources revealed that P1 likely had a *Streptococcus* infection, P3 had *Escherichia coli* infection*,* and P5 had EBV infection. P2 had an unknown infection and high fever a few days before the sample was collected and sent for testing. However, P4 did not display any infection symptoms at the time of diagnosis (Table 1). Overall, patients with FXIII deficiency with a pathogenic infection are likely more susceptible to developing autoantibodies. However, due to the limited number of patients, in vivo mouse models were used for further studies.

Many hemolytic and thrombotic diseases, including paroxysmal nocturnal hemoglobinuria, autoimmune hemolytic anemia, atypical hemolytic uremic syndrome (aHUS), and DIC, are associated with complement dysfunction (15, 16). Evidently, the complement and coagulation systems are evolutionary related enzymatic cascades in the blood circulation, which are cross-activated and further influence host immune functions after tissue injury (17, 18). Therefore, we investigated whether FXIII deficiency is associated with complement dysfunction and further influences host immune responses against pathogenic infections. The data showed that P1–P5 had decreased complement activation, whereas P6–P11 had normal complement activation (Figure 1, C–E, and Supplemental Figure 1, C and D). Further analysis revealed that regarding alternative pathway (AP) activation, P1, with the highest autoantibody level, had 89% less membrane attack complex (MAC) formation and 93% less C3b/iC3b deposition than healthy control individuals. Similarly, P2–P5 with lower autoantibody levels displayed 85%, 73%, 49%, and 70% less MAC formation and 90%, 77%, 53%, and 69% less C3b/iC3b deposition, respectively, compared with the healthy donors. However, 4 patients with nonimmune deficiency and 2 patients with congenital deficiency with no detectable autoantibodies had normal complement activation (Figure 1, C and D). Similar patterns of MAC formation and C3b/iC3b deposition were observed for both the lectin pathway (LP) and classical pathway (CP) activation in all patients, although the differences of LP and CP activation from the healthy donors were less pronounced (Supplemental Figure 1, C and D). The complement activation pattern in all patients was confirmed with a hemolytic assay (Figure 1E).

Collectively, these findings indicate patients with autoimmune FXIII deficiency, but not those with undetectable autoantibodies, exhibit decreased complement activation. Because this decreased complement activation is likely consistent with complement depletion or inhibition, the levels of the central complement component C3 and complement inhibitors, including factor H (FH), C1 inhibitor (C1INH), C4b binding protein (C4BP), and vitronectin, in the plasma of patients and healthy donors were analyzed by Western blot (WB) using transferrin as an internal reference. FH, C1INH, C4BP, and vitronectin were detected with variable levels in the plasma of all patients, but without substantial differences from those in the plasma of healthy donors (Supplemental Figure 1, E and F), indicating that decreased complement activation in P1–P5 was not attributed to high levels of complement inhibitors. However, complement C3 levels were substantially lower in P1–P5 than in P6–P11 and the healthy donors. Low complement C3 levels could be caused by overactivation of the complement system in vivo or autoantibody-mediated neutralization or rapid clearance of C3 from blood circulation. To determine this, C3a and C5a levels, markers of the complement activation state, in the plasma of patients were quantified using WB and ELISA. The results revealed trace amounts of C3a and C5a in most patients and healthy donors, with even lower C3a and C5a levels in P1, P3, P4, P5, and P11 than in normal human serum (NHS) (Supplemental Figure 1, E and F). These results suggest that lower C3 levels in P1–P5 were not attributed to complement overactivation-mediated depletion or consumption. However, the reason for decreased complement activation and lower C3 levels in patients with autoimmune FXIII deficiency requires further investigation.

### A combined FXIII-C3 autoantibody was identified in patients with autoimmune FXIII deficiency, which, by neutralizing C3, inhibited complement activation.

**Figure 2:**
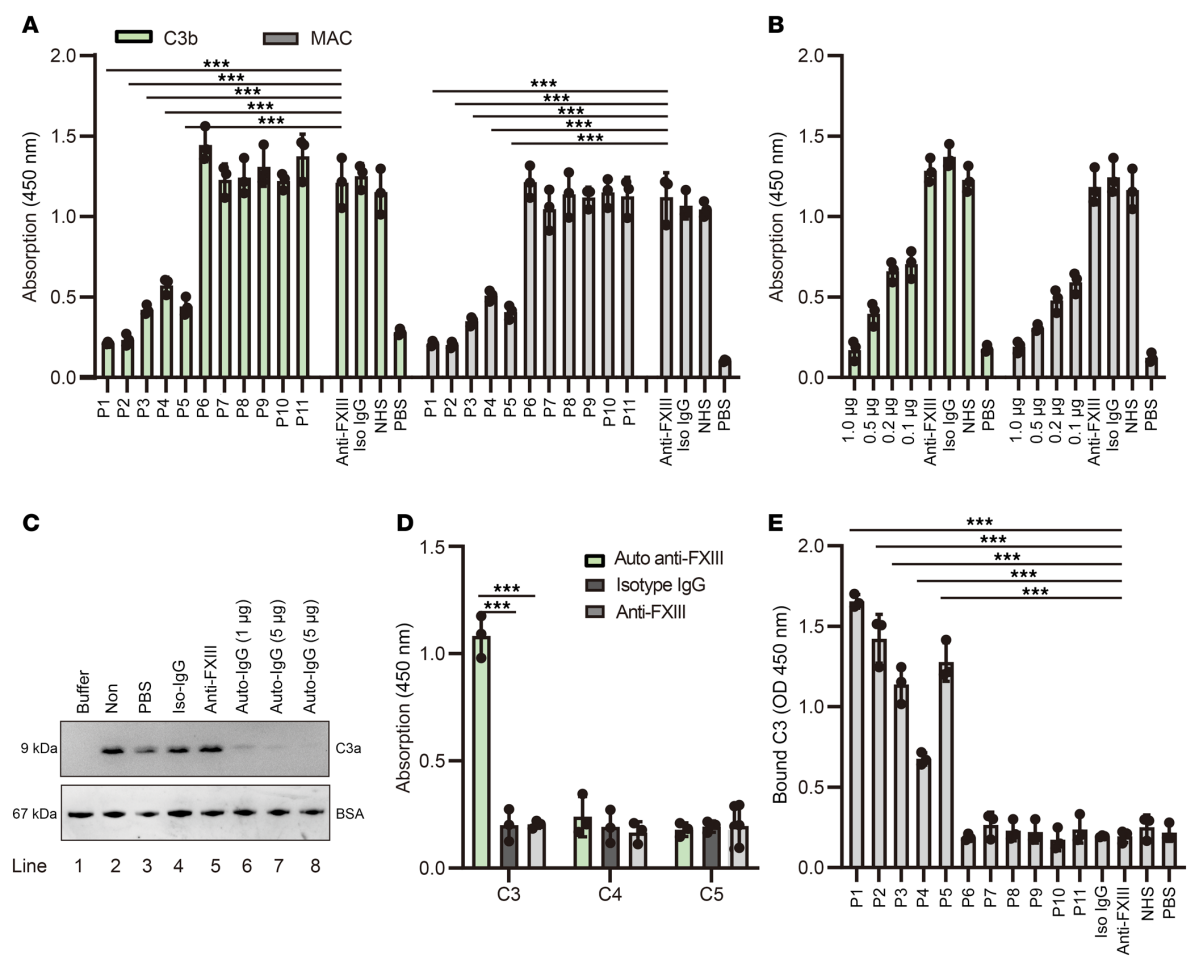
A combined FXIII-C3 autoantibody was identified in patients with autoimmune FXIII deficiency, which, by neutralizing C3, inhibited complement activation. (**A**) Effects of anti–FXIII autoantibody on complement activation. Autoantibody was first fished from different patients’ plasma using precoated FXIII-A, then incubated with NHS. These pretreated NHS samples was further added to LPS-coated plates for complement activation analysis. (**B**) Dose-dependent effects of purified anti–FXIII autoantibody on complement activation. (**C**) Anti–FXIII autoantibody–mediated blockage of C3 cleavage by preformed C3 convertase. BSA was used as loading control. A representative blot (of 4) is shown. (**D**) Binding of purified anti–FXIII autoantibody to complement C3, C4, or C5. (**E**) Existence of combined FXIII-C3 autoantibodies in patients with autoimmune FXIII deficiency. Isotype human IgG (Iso-IgG) and commercial anti-FXIII were used as controls. Data are shown as mean ± SD. One-way ANOVA was used for statistical analysis. ***P < 0.001. Non, C3 sample was not pre-treated; PBS, C3 sample was pre-incubated with PBS. anti-FXIII, commercial anti-FXIII antibody (Proteintech, catalog 66325-1-Ig).

Based on these data, patients with autoimmune FXIII deficiency, but not those with undetectable autoantibodies, had reduced complement activation, and the decreased level was likely positively correlated with the anti–FXIII autoantibody levels. We hypothesized that anti–FXIII autoantibodies were the main determinants interfering with complement activation in patients. To validate this, the NHS was initially pretreated with the anti–FXIII autoantibody “fished” by immobilized FXIII from the plasma of different patients and subsequently added to an LPS-coated ELISA plate to quantify complement activation by analyzing C3b deposition and MAC formation (isotype IgG and commercial anti-FXIII antibody [Proteintech, catalog 66325-1-Ig] were used as controls). Serum preincubated with anti–FXIII autoantibody fished from P1 had approximately 82% less C3b deposition and 81% less MAC formation than the serum pretreated with commercial anti-FXIII. Similarly, serum pretreated with anti–FXIII autoantibody fished from P2, P3, P4, and P5 had 81%, 65%, 53%, and 64% less C3b formation, as well as 82%, 69%, 55%, and 64% less MAC deposition, respectively (Figure 2A). However, serum pretreated with nonspecific IgG fished from P6–P11 had similar levels of C3b deposition and MAC formation as serum pretreated with commercial anti-FXIII and isotype IgG. When further compared with the untreated NHS, no statistical difference was observed in the serum pretreated with commercial anti-FXIII and isotype IgG (Figure 2A). These data indicate that anti–FXIII autoantibodies developed in patients with autoimmune FXIII deficiency inhibited complement activation.

To further verify the anti–FXIII autoantibody–mediated inhibitory effect on complement activation, the anti–FXIII autoantibody was first purified from the plasma of P1 using an affinity column. The quality of these purified autoantibodies was analyzed using Coomassie staining and “Far Western” blot. Both purified anti–FXIII autoantibodies and isotype IgG had greater than 90% purity (Supplemental Figure 2A), and purified anti–FXIII autoantibodies, but not isotype IgG, specifically bound to FXIII antigen (Supplemental Figure 2A). To exclude any possible complement inhibitors or C3 present in the purified anti–FXIII autoantibody, WB was performed using specific antibodies against complement FH, C1INH, C3, C4BP, and vitronectin. None of the complement inhibitors or proteins were present in the purified autoantibodies (Supplemental Figure 2B).

Next, NHS was preincubated with different amounts of purified autoantibodies using isotype IgG and commercial anti–FXIII as controls, and complement activation was analyzed. Our data demonstrated that the purified autoantibody dose-dependently inhibited complement activation, as evidenced by levels of C3b deposition and MAC formation that decreased along a gradient. When used at 1 μg, the purified anti–FXIII autoantibody inhibited C3b deposition by 87% and MAC formation by 85% (Figure 2B), compared with the commercial anti–FXIII control. We note that the commercial anti-FXIII and anti-C3 do not cross-react with C3 or FXIII (Supplemental Figure 2C). Collectively, these data further prove that anti–FXIII autoantibody dose-dependently inhibited complement activation, particularly at the C3 level of the complement cascade.

To further illustrate the mechanism by which this autoantibody disrupts complement activation at the C3 level, we first attempted to build a C3 convertase on a zymosan surface, using purified complement components, then we quantified it by flow cytometry using an anti–human Bb antibody. The results suggested the successful construction of C3 convertase on the zymosan surface (Supplemental Figure 2D). We then analyzed whether the anti–FXIII autoantibody affects C3 convertase formation by binding to complement components, such as factors B, D, and P. To investigate this, the purified anti–FXIII autoantibody was preincubated with these complement factors and then mixed with C3b opsonized particles. After further incubation and extensive washing, the level of C3 convertase formation on zymosan particles was again determined by flow cytometry. The results revealed that the anti–FXIII autoantibody did not affect C3 convertase formation (Supplemental Figure 2E).

Afterward, we investigated whether anti–FXIII autoantibody prevents C3 cleavage by a preformed C3 convertase. To achieve this, the C3 convertase was again constructed on a zymosan surface. Meanwhile, C3 was preincubated with the different amounts of purified autoantibody, PBS, isotype human IgG, or commercial anti–FXIII, then added to the preassembled C3 convertase. After incubation, C3a generation was determined by WB. The results revealed that purified anti–FXIII autoantibody did dependently inhibited C3 cleavage by preformed C3 convertase, as evidenced by a dose-dependent decrease in C3a levels (Figure 2C, upper panel, lines 6–8), compared with nontreated C3, C3 pretreatment with PBS, isotype human IgG, or commercial anti-FXIII (Figure 2C, upper panel, lines 2–5). Because the incubation buffer contained BSA, the loading control was indicated by the BSA level in each sample (Figure 2C, lower panel). Collectively, these data indicated that by forming a complex with C3, anti–FXIII autoantibodies blocked C3 cleavage and C3a release, thereby efficiently inhibiting complement activation.

These observed effects of anti–FXIII autoantibodies on C3 cleavage suggest anti–FXIII autoantibodies also complex C3 in addition to binding to FXIII. To prove this, purified anti–FXIII autoantibodies, isotype IgG, and commercial anti-FXIII were coated on the wells, and C3, C4, or C5 were added for further incubation. Therefore, the binding intensity was quantified using specific antibodies. The results demonstrated that the purified anti–FXIII autoantibody, but not isotype IgG or commercial anti-FXIII, specifically bound to C3, but not to C4 or C5 (Figure 2D). These findings highlight that combined FXIII-C3 autoantibodies may exist in patients with autoimmune FXIII deficiency.

To further prove the presence of the combined FXIII-C3 autoantibody in patients with autoimmune FXIII deficiency, which binds both FXIII and C3, FXIII was first coated on the ELISA plate, and patients’ plasma and normal human plasma (NHP) were added and incubated to capture anti–FXIII autoantibodies from the plasma. Purified C3 was then added to the wells for further incubation. Finally, bound C3 was detected using a specific goat anti–human C3 antibody. The results showed that autoantibodies fished by precoated FXIII-A from P1–P5 specifically bound C3. However, no signal was detected for P6–P11, who had no autoantibodies, and healthy donors. Upon further comparison, P1, P2, P3, and P5, who had higher autoantibody levels, binding intensity was much stronger to C3 than P4 (Figure 2E).

Taken together, a combined FXIII-C3 autoantibody exists in patients with autoimmune FXIII deficiency, and it binds both FXIII and C3. By forming a complex with C3, this autoantibody blocks C3 cleavage and mediates rapid clearance of C3 from the blood circulation, thereby interfering with complement activation at the C3 level. Because complement activation serves as a central immune surveillance against invading pathogens, autoantibody-mediated complement inhibitory effects likely increase host susceptibility to pathogenic infection.

### Autoimmune mice with FXIII-C3-deficiency had severe pathogenic Candida albicans infections by modulating complement and T cell activation.

**Figure 3:**
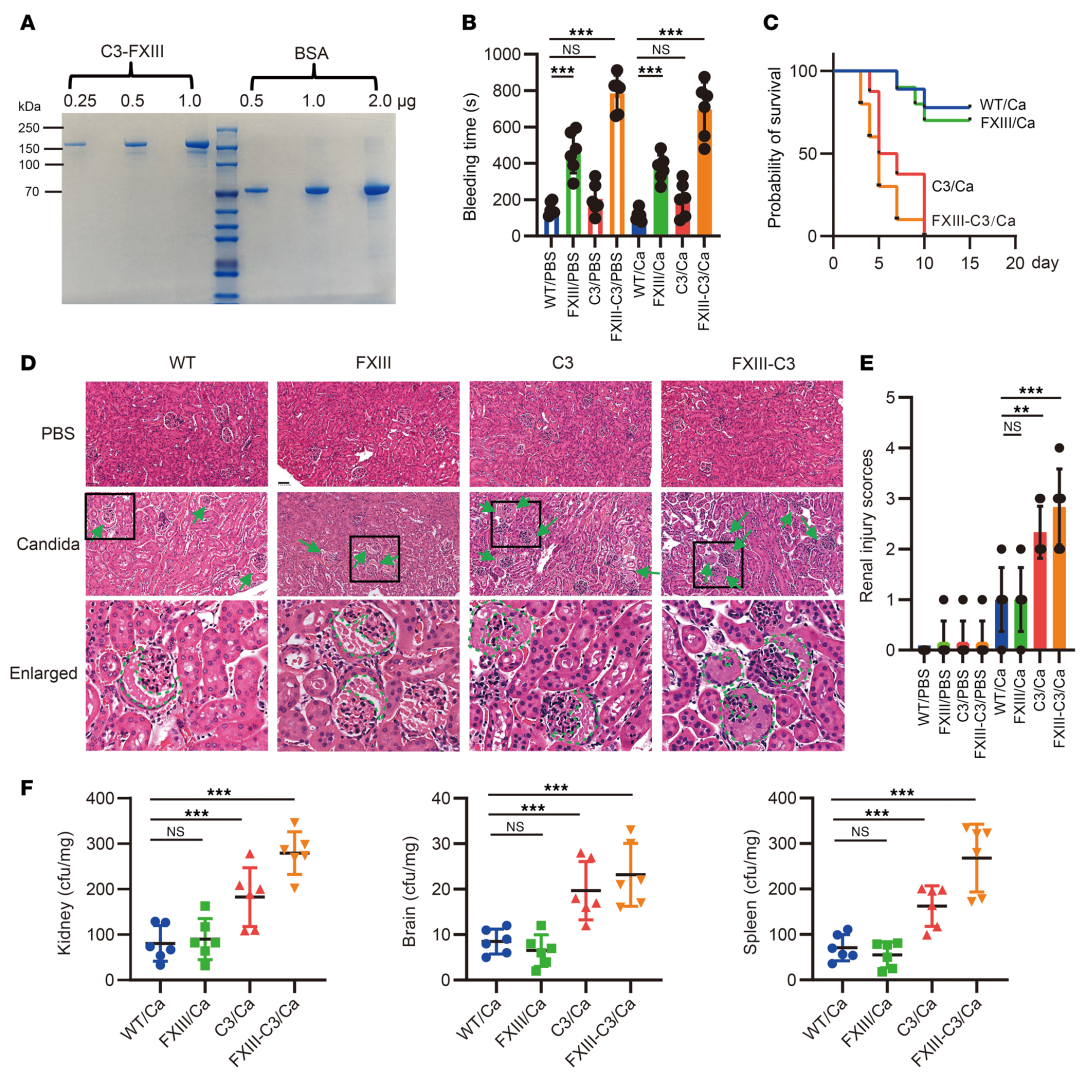
Autoimmune FXIII-C3–deficient mice had severe pathogenic infections with *C*. *albicans*. (**A**) Coomassie staining of fusion protein C3-FXIII. The left side of the gel contain different amounts of C3-FXIII fusion protein (0.25, 0.5, and 1 μg), the right side contains different amount of the control protein BSA (0.5, 1, and 2 μg). (**B**) Tail bleeding time of different groups of mice. (**C**) Survival rate of different groups of mice. A dose of 1 × 10^5^ *C*. *albicans* per mouse was applied (*n* = 10 per group). The infection period was 15 days. (**D**) H&E staining for kidney pathology upon fungal infection. Representative images out of 6 are shown. (**E**) Renal injury score evaluated by images of H&E staining. (**F**) Fungal burdens of different organs. For these in vivo experiments, C57BL/6J mice were first immunized by PBS, FXIII, C3, or C3-FXIII, generating different autoimmune-deficient mice, simply named WT, FXIII, C3, and FXIII-C3. These mice were further challenged with *C*. *albicans,* generating 8 groups of mice, which were classified as WT/Ca and WT/PBS, FXIII/Ca and FXIII/PBS, C3/Ca and C3/PBS, FXIII-C3/Ca and FXIII-C3/PBS. For (**B**) and (**D**–**F**), 5 × 10^4^ *C*. *albicans* per mouse was applied (*n* = 6 per group). Infection time is 7 days. Data are shown as mean ± SD; 1-way ANOVA was used for statistical analysis. The survival rate of mice was analyzed by a Kaplan-Meier log-rank test. Scale bar: 100 μm. **P < 0.01, ***P < 0.001.

To determine whether the combined FXIII-C3 autoantibody increased host susceptibility to pathogenic infection by modulating complement activation and complement-mediated adaptive immune responses, a proper model of autoimmune FXIII–C3 deficiency needs to be generated. Application of a 7-phage-display peptide library together with ELISA and Far Western blot found that only in the presence of thrombin and Ca^2+^ did FXIII bind to C3 via the N-terminal epitopes FTAPYPR and LTPFVHL (highlighted in red in Supplemental Figure 3D). Based on this information, fusion protein C3-FXIII was constructed by linking C-terminus of C3-α chain with N-terminus of FXIII-A with a flexible linker (GGGGS)_3_ inserted in the middle and then was expressed and purified (Figure 3A).

Purified C3–FXIII fusion protein was then intramuscularly injected into mice to generate autoimmune models of FXIII-C3 deficiency; FXIII, C3, and PBS were applied as controls (Supplemental Figure 4A). These 4 groups of mice were named as WT, FXIII, C3, and FXIII-C3. At days –8, –6, and –2, a decrease in FXIII antigen levels was detected in the FXIII and FXIII-C3 groups, and a decrease in C3 levels was detected in the C3 and FXIII-C3 groups (Supplemental Figure 4B), whereas an increase in combined autoantibodies with bispecificity against FXIII and C3 was detected only in the FXIII-C3 group of mice. However, monospecific autoantibodies against FXIII or C3 were detected in the FXIII or C3 group of mice, respectively (Supplemental Figure 4C), indicating that autoimmune responses were triggered, and that mouse models mimicking the conditions of patients with autoimmune FXIII deficiency were successfully generated.

Second, the 4 groups of mice were further challenged with *Candida albicans* or PBS intravenously. Therefore, 8 groups of mice were generated and classified as WT/Ca, WT/PBS, FXIII/Ca, FXIII/PBS, C3/Ca, C3/PBS, FXIII-C3/Ca, and FXIII-C3/PBS. Tail bleeding time, survival rate, tissue damage, and fungal burden were then analyzed. The results demonstrated that bleeding time was prolonged in FXIII and FXIII-C3 groups of mice, but not the C3 group of mice, both before and after infection compared with WT controls (Figure 3B), and this was associated with autoantibody-mediated neutralization of FXIII. Monitoring of the survival rate demonstrated that 20% of the mice in the FXIII-C3/Ca group died on day 3, 40% died on day 4, and all mice had died by day 10 after infection. The C3/Ca group of mice behaved similarly to FXIII-C3/Ca mice, with 40% of mice dying on day 5 and all mice dying by day 10. In contrast, 30% of WT/Ca and 40% of FXIII/Ca mice died on day 10, whereas 70% of WT/Ca and 60% of FXIII/Ca mice survived until day 15 after infection (Figure 3C). Additionally, when infected, FXIII-C3/Ca and C3/Ca groups had much heavier spleen weights than did WT/Ca and FXIII/Ca groups of mice, whereas the body weight and weights of organs remained equal among individual mice (Supplemental Figure 4, D–F).

H&E staining showed that severe hyperplasia of the glomerular wall epithelium was commonly observed in the kidneys of the FXIII-C3/Ca group of mice, leading to the formation of crescents marked in Figure 3D with a green arrow in the general overview and green dashed box in the magnified images, whereas moderate hyperplasia of the glomerular wall epithelium was detected in the kidneys of C3/Ca mice. In contrast, mild hyperplasia of the glomerular wall epithelium was observed in the kidneys of WT/Ca and FXIII/Ca mice (Figure 3D). Renal injury scores were evaluated based on the large field of slide view; these are shown in Figure 3E. Moreover, a substantially higher fungal load was identified in the FXIII-C3/Ca and C3/Ca groups than in the WT/Ca group, whereas a similar fungal load was detected in the FXIII/Ca and WT/Ca groups of mice (Figure 3F and Supplemental Figure 4G), indicating that severe *C*. *albicans* infection was initiated in autoimmune FXIII-C3 and C3-deficient mice, mainly associated with C3 inactivation or deficiency.

**Figure 4:**
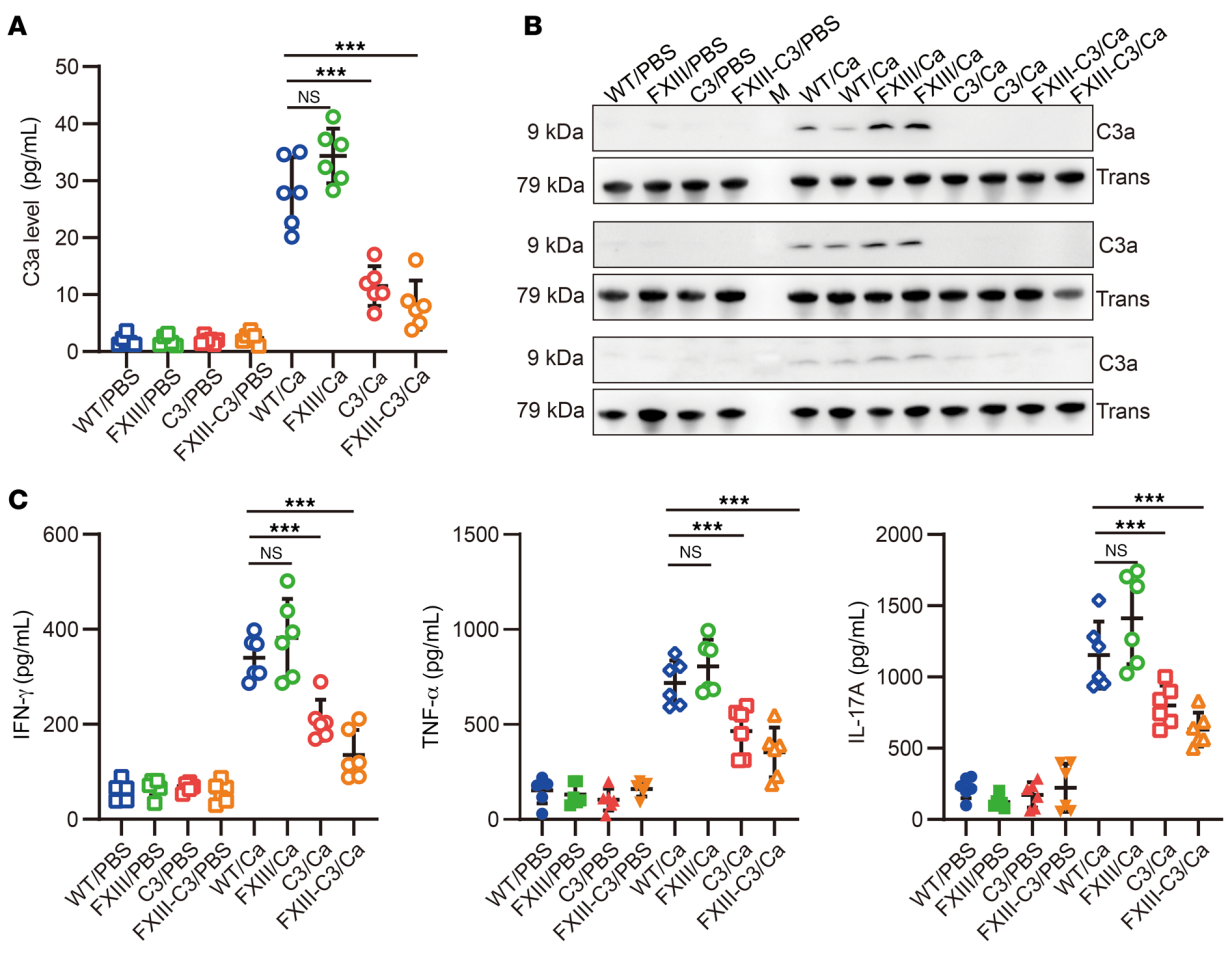
Autoimmune FXIII-C3–deficient mice had defective complement activation and pro-inflammatory cytokine release. (**A** and **B**) C3a release in mice plasma detected by ELISA and WB. Transferrin was used as a loading control. A representative blot (out of 4) is shown. (**C**) Inflammatory cytokine release in mice plasma at day 7 after infection detected by ELISA kit. In the in vivo infection model, WT, FXIII, C3, and FXIII-C3 groups of mice were intravenously injected with *C*. *albicans* (5 × 10^4^/mouse) or PBS for 7 days, 8 groups of mice were classified as WT/Ca and WT/PBS, FXIII/Ca and FXIII/PBS, C3/Ca and C3/PBS, or FXIII-C3/Ca and FXIII-C3/PBS (*n* = 6 per group). Data are shown as mean ± SD; 1-way ANOVA was used for statistical analysis. ***P < 0.001.

**Figure 5:**
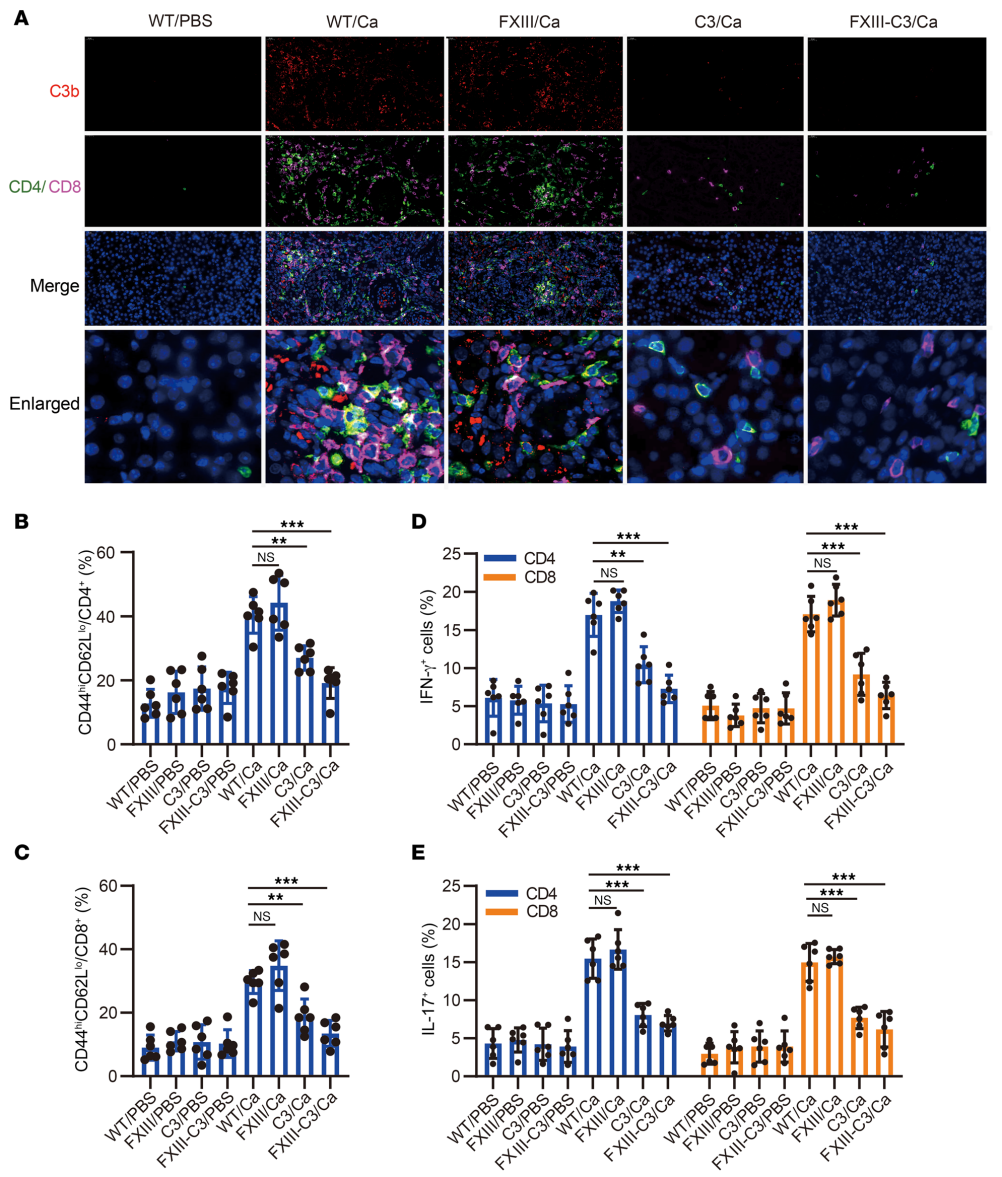
Autoimmune FXIII-C3–deficient mice had defective complement and T cell activation. (**A**) C3b/iC3b deposition and T cell infiltration in kidney were analyzed by immunofluorescent staining. Representative images (out of 6) are shown. (**B** and **C**) CD4 and CD8 T cell activation upon fungal infection. T cell activation was quantified by the CD44^high^CD62L^low^ portion of CD4^+^ and CD8^+^ T cells via flow cytometry. (**D** and **E**) Portion of IFN-γ^+^ or IL-17^+^ CD4 and CD8 T cells analyzed by flow cytometry. In the in vivo infection model, WT, FXIII, C3, and FXIII-C3 groups of mice were intravenously injected with *C*. *albicans* (5 × 10^4^/mouse) or PBS for 7 days; 8 groups of mice were classified as WT/Ca and WT/PBS, FXIII/Ca and FXIII/PBS, C3/Ca and C3/PBS, or FXIII-C3/Ca and FXIII-C3/PBS (*n* = 6 per group). Data are shown as mean ± SD, 1-way ANOVA was used for statistical analysis. Scale bar: 40 μm, original magnification, x400. **P < 0.01, ***P < 0.001.

To further analyze whether the uncontrolled severe *C*. *albicans* infection was associated with complement C3 inactivation and defects in downstream immune responses, C3a release, cytokine release, C3b deposition, T cell infiltration, and T cell activation were monitored on day 7 after infection, following the indicated experimental schedule (Supplemental Figure 5A). The results revealed that almost no or just a trace of C3a was detected in the FXIII-C3/Ca and C3/Ca groups of mice, which was much less than that detected in the WT/Ca and FXIII-Ca groups (Figure 4, A and B). Meanwhile, lower levels of IFN-γ, TNF-α, and IL-17A were also detected in the serum of FXIII-C3/Ca and C3/Ca groups of mice compared with those in WT/Ca and FXIII/Ca mice (Figure 4C). Subsequently, immunofluorescence staining demonstrated that the FXIII-C3/Ca and C3/Ca groups of mice had reduced C3b/iC3b deposition and less CD4^+^/CD8^+^ T cell infiltration in the kidney compared with WT/Ca and FXIII-Ca groups of mice (Figure 5A). Further flow cytometry analysis of T cell activation in the spleen revealed that the FXIII-C3/Ca and C3/Ca groups of mice had fewer CD44^high^ CD62^low^ portions among CD4^+^ and CD8^+^ T cells than did WT/Ca and FXIII/Ca groups of mice (Figure 5, B and C, Supplemental Figure 5B). Meanwhile, lower levels of IFN-γ and IL-17–secreting CD4^+^ and CD8^+^ T cells were also identified in the FXIII-C3/Ca and C3/Ca groups of mice compared with those in the WT/Ca and FXIII-Ca groups (Figure 5, D and E, and Supplemental Figure 5C). These data indicate that less complement and T cell activation were initiated in autoimmune FXIII–C3 and C3-deficient mice.

### Pathogenic C. albicans infections further induced autoantibody generation.

**Figure 6:**
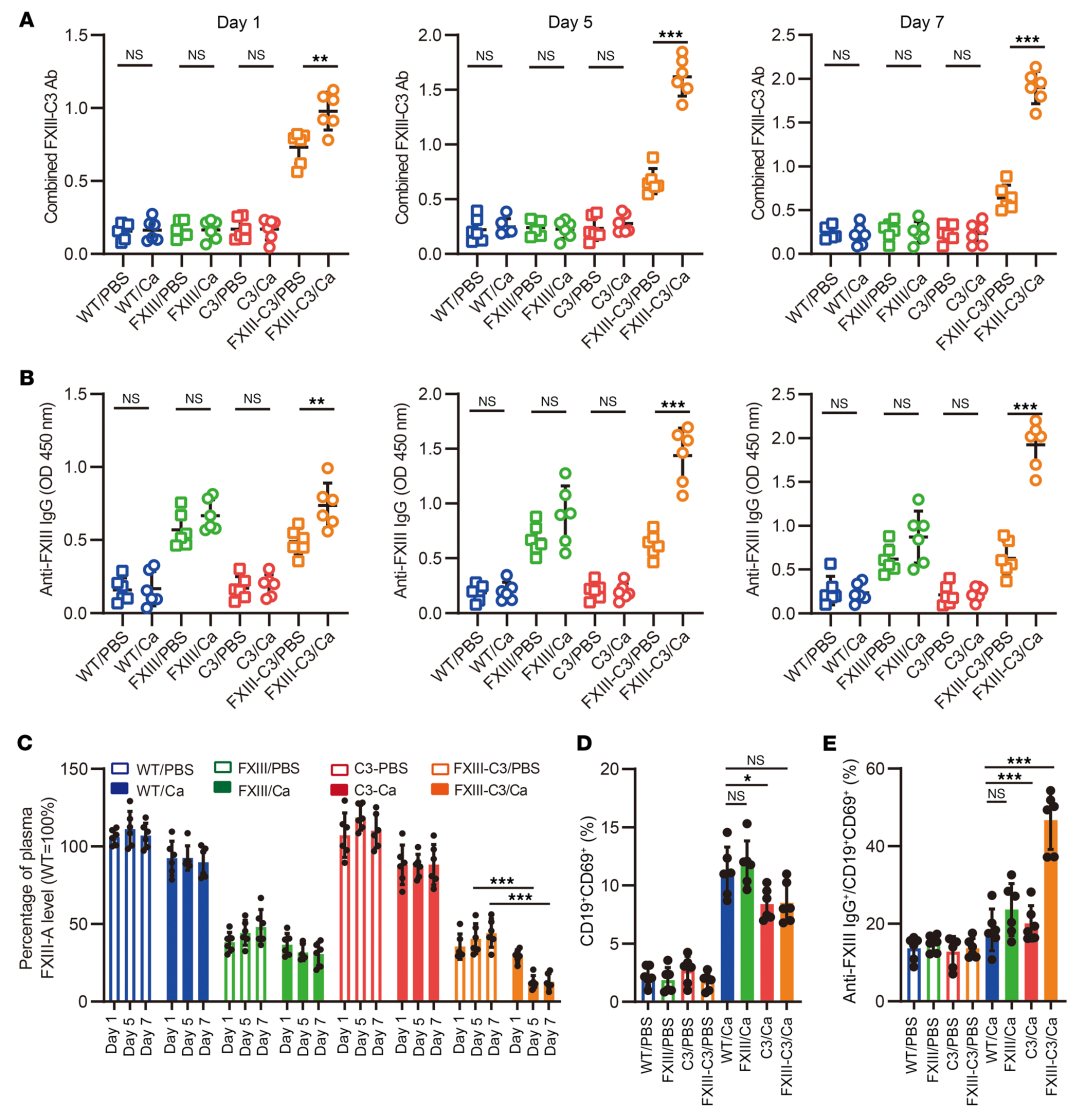
Pathogenic *C*. *albicans* infections further induced autoantibody generation in mice. Combined FXIII-C3 autoantibody (**A**), and anti–FXIII autoantibody (**B**) in 8 groups of mice on days 1, 5, and 7 after infection were detected by ELISA. (**C**) FXIII-A antigen level in 8 groups of mice on days 1, 5, and 7 after infection. The FXIII-A antigen level of WT/PBS group on day 1 was set as 100%, the other samples were calculated accordingly. (**D**) Total B cell activation. Cells were isolated from the spleen and analyzed by flow cytometry. B cell activation is shown by the percentage of CD19^+^CD69^+^ B cells. (**E**) Autoreactive B cell activation. Again, cells were isolated from the spleen and analyzed by flow cytometry. Anti–FXIII IgG^+^ autoreactive B cells activation is presented by the percentage of anti–FXIII IgG^+^ B subgroup among CD19^+^CD69^+^ B cells. In this in vivo experimental settings, WT, FXIII, C3, and FXIII-C3 groups of mice were intravenously injected with *C*. *albicans* (5 × 10^4^/mouse) or PBS, and 8 groups of mice were classified as WT/Ca and WT/PBS, FXIII/Ca and FXIII/PBS, C3/Ca and C3/PBS, or FXIII-C3/Ca and FXIII-C3/PBS (*n* = 6 per group). Data are shown as mean ± SD. An unpaired 2-tailed Student’s *t* test was used for statistical analysis for **A** and **B**. One-way ANOVA was used for statistical analysis for **C**–**E**. *P < 0.05, **P < 0.01, ***P < 0.001.

In a recent study on COVID-19, Lichter et al. (13) reported that 74% of patients in an ICU with COVID-19 had decreased and defective FXIII activity, and that FXIII levels gradually decreased during hospitalization with frequent severe bleeding. Moreover, our clinical data demonstrated that patients with autoimmune FXIII deficiency who had pathogenic infections had much higher levels of combined FXIII-C3 autoantibodies, along with much lower levels and activity of FXIII (Table 1). Collectively, these data suggested that pathogenic infections likely further induce autoantibody production in autoimmune FXIII-deficient conditions. To determine this, the different groups of mice we generated were again applied. Upon intravenous infection with *C*. *albicans,* blood samples were collected from different mouse groups on days 1, 5, and 7 after infection. Monospecific autoantibodies against FXIII or C3 and combined autoantibodies with bispecificity against FXIII and C3 were then analyzed using sandwich ELISA. On day 1, the combined autoantibody was detected only in the FXIII-C3 group of mice both before and after infection, whereas monospecific autoantibodies against FXIII were detected in the FXIII mice and monospecific autoantibodies against C3 were detected in the C3 mice. However, no autoantibodies were detected in WT mice (Figure 6, A and B, and Supplemental Figure 6A). On days 5 and 7, as the infection progressed, the FXIII-C3/Ca group of mice developed substantially higher levels of combined FXIII-C3 autoantibodies compared with the FXIII-C3/PBS group, and substantially higher levels of monospecific anti–C3 autoantibodies also were detected in the C3/Ca group than in the C3/PBS group (Supplemental Figure 6A). In contrast, the number of monospecific anti–FXIII autoantibodies was slightly increased in FXIII/Ca compared with the FXIII/PBS group of mice, although the difference was statistically nonsignificant, whereas the numbers of autoantibodies in the noninfected groups were almost equal to those on day 1. Overall, autoantibody generation was triggered upon uncontrolled *C*. *albicans* infection, such as combined autoantibodies against FXIII and C3 and monospecific autoantibodies against C3.

Quantification of FXIII-A levels demonstrated that either C3-FXIII fusion protein or FXIII immunization resulted in approximately 70% removal of FXIII (Supplemental Figure 4B). Further analysis revealed that on day 1 after infection, FXIII levels in all 4 groups of mice with *Candida* infection were slightly reduced compared with those in the noninfected groups (Figure 6C), which is likely associated with the acute consumption of FXIII upon *C*. *albicans* infection. Interestingly, only in the FXIII-C3/Ca group did FXIII levels decline further on day 5 after infection, and greater than 95% of FXIII was consumed on day 7 after infection. A slight decrease in FXIII level was also observed in FXIII/Ca group of mice as the infection progressed, although the difference was statistically nonsignificant. Overall, these data indicated that FXIII levels progressively decline upon *C*. *albicans* infection in autoimmune FXIII-C3 deficiency.

Analysis of B cell activation revealed that the frequency of CD19^+^CD69^+^ B cells representing B cell activation was increased in all 4 fungus-infected groups, with a slightly higher level in the WT/Ca and FXIII/Ca groups than in the FXIII/Ca and FXIII-C3/Ca groups (Figure 6D and Supplemental Figure 6B). However, further quantification of anti–FXIII IgG^+^ subgroup B cell frequency among activated B cells revealed a substantially higher percentage of anti–FXIII IgG^+^ autoreactive B cells specifically in the FXIII-C3/Ca group, compared with those in the WT/Ca, FXIII/Ca, and C3/Ca groups (Figure 6E and Supplemental Figure 6C), meaning that although B cells were activated in all 4 *Candida*-infected groups, more anti–FXIII IgG^+^ autoreactive B cells were activated only in the FXIII-C3/Ca group of mice. These results indicated that *C*. *albicans* infection further amplifies autoimmune signals in mice with autoimmune FXIII-C3 deficiency, resulting in the progressive release of combined FXIII-C3 autoantibodies.

### Pathogenic C. albicans infection–promoted autoantibody production was mediated by platelet-released CD40L.

**Figure 7:**
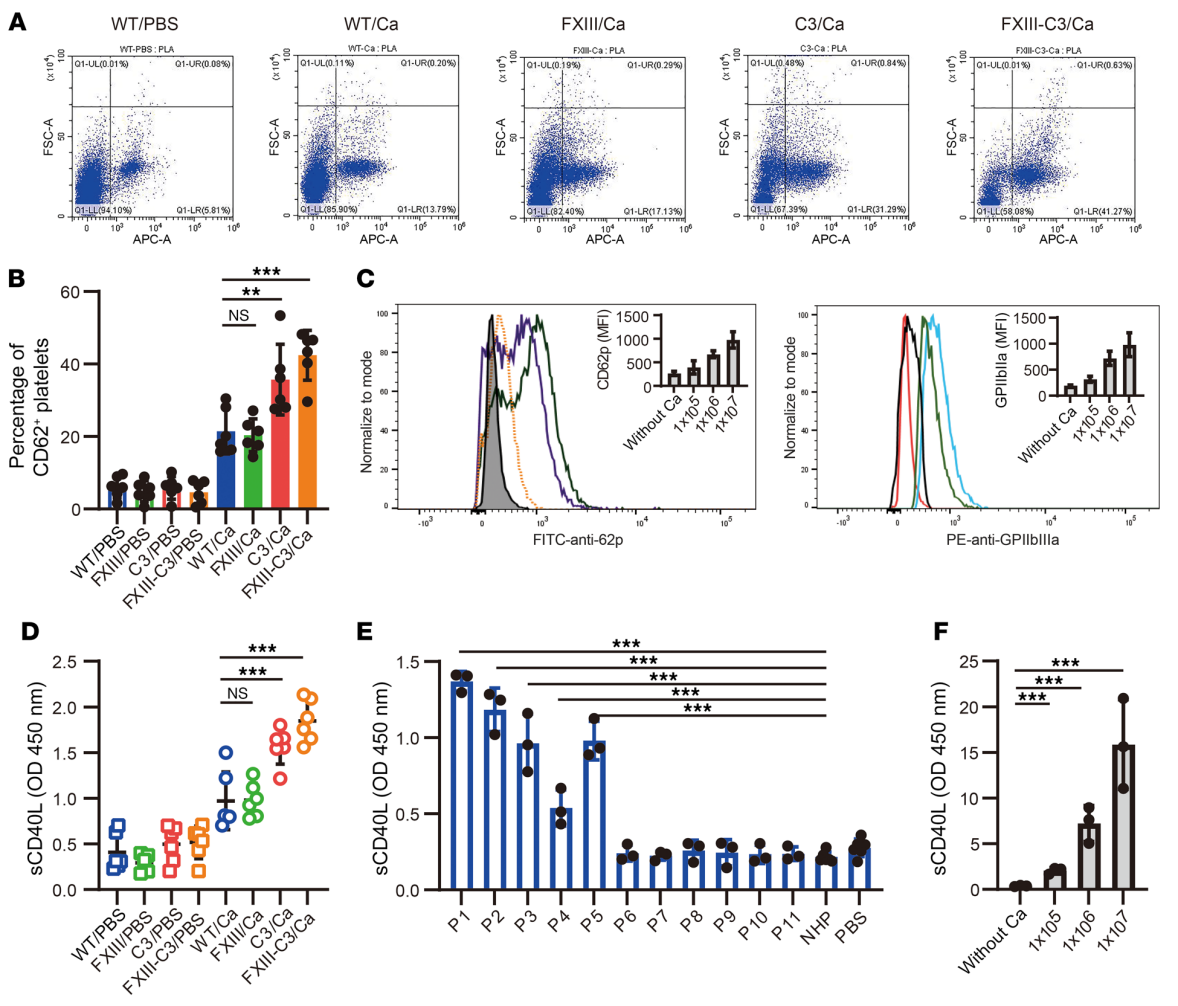
Pathogenic *C*. *albicans* infection triggered platelet activation and CD40L release. (**A** and **B**) Quantification of platelets’ activation in 8 different groups of mice via CD62p surface expression analyzed by flow cytometry. A representative dot plot (**A**) and statistical chart of activated platelets (**B**) are shown. (**C**) Platelet activation upon in vitro co-culture of platelets with *C*. *albicans*. CD62p expression (left) and GPIIbIIIa expression (right) on platelet surfaces were analyzed by flow cytometry upon in vitro co-culture of platelets (1 × 10^7^/sample) with different doses of *C*. *albicans* (1 × 10^5^, 1 × 10^6^ and 1 × 10^7^/sample) in the presence of Gly-Pro-Arg-Pro (Pefa 6003) to prevent platelet aggregation. (**D**) Soluble CD40L levels in plasma of 8 groups of mice on day 7 after infection. (**E**) Soluble CD40L levels in plasma of patients with FXIII deficiency and in healthy control donors. (**F**) CD40L release in the platelet-*C*. *albicans* co-culture supernatant. Data are shown as mean ± SD; 1-way ANOVA was used for statistical analysis. **P < 0.01, ***P < 0.001. FSC-A, forward scatter area; PLA, proximity ligation assay; APC-A, allophycocyanin area.

Wang et al. reported that platelet-related CD40L release plays a vital role in B cell activation (10). To determine the molecular basis of fungal infection–amplified combined FXIII-C3 autoantibody production, platelet activation was first monitored in the 8 groups of mice using flow cytometry. The results indicated FXIII-C3/Ca and C3/Ca groups of mice had, respectively, approximately 41.27% and 31.29% activated platelets on day 7 after infection, whereas WT/Ca and FXIII/Ca groups had 13.79% and 17.13% activated platelets, respectively (Figure 7, A and B). These higher platelet-activation rates observed in FXIII-C3/Ca and C3/Ca groups of mice are likely attributed to uncontrolled *C*. *albicans* infection.

To further confirm *C*. *albicans–*mediated platelet activation, in vitro co-culture experiments were performed. Briefly, different doses of *C*. *albicans* were incubated with platelets isolated from the blood of healthy donors, and the platelet surface CD62p and GPIIbIIIa levels were quantified. The results revealed that CD62p and GPIIbIIIa expression on the platelet surface was upregulated. The higher the amount of *C*. *albicans* added, the higher the expression levels of CD62p and GPIIbIIIa were (Figure 7C), indicating platelet activation was induced upon *C*. *albicans* stimulation in a dose-dependent manner.

Subsequently, platelet-related CD40L release upon platelet activation was quantified using ELISA in the mice we generated, as well as samples from patients with FXIII deficiency and *Candida*–platelet co-cultures. The results demonstrated that all 4 fungus-infected groups (WT/Ca, FXIII/Ca, C3/Ca, and FXIII-C3/Ca) had higher plasma CD40L levels compared with the uninfected groups (WT/PBS, FXIII/PBS, C3/PBS, and FXIII-C3/PBS). Furthermore, substantially higher CD40L levels were detected in FXIII-C3/Ca and C3/Ca groups than in WT/Ca and FXIII/Ca groups (Figure 7D). Meanwhile, P1 had the highest plasma CD40L levels, followed by P2, P3, P5, and P4. However, P6–P11 had comparable CD40L levels to the healthy donors (Figure 7E). Further quantification of platelet-related CD40L release in the Candida–platelet co-culture supernatant revealed that *C*. *albicans* dose dependently induced platelets to release CD40L (Figure 7F). These findings indicated platelet activation and platelet-related CD40L release were induced upon *C*. *albican*s infection.

**Figure 8:**
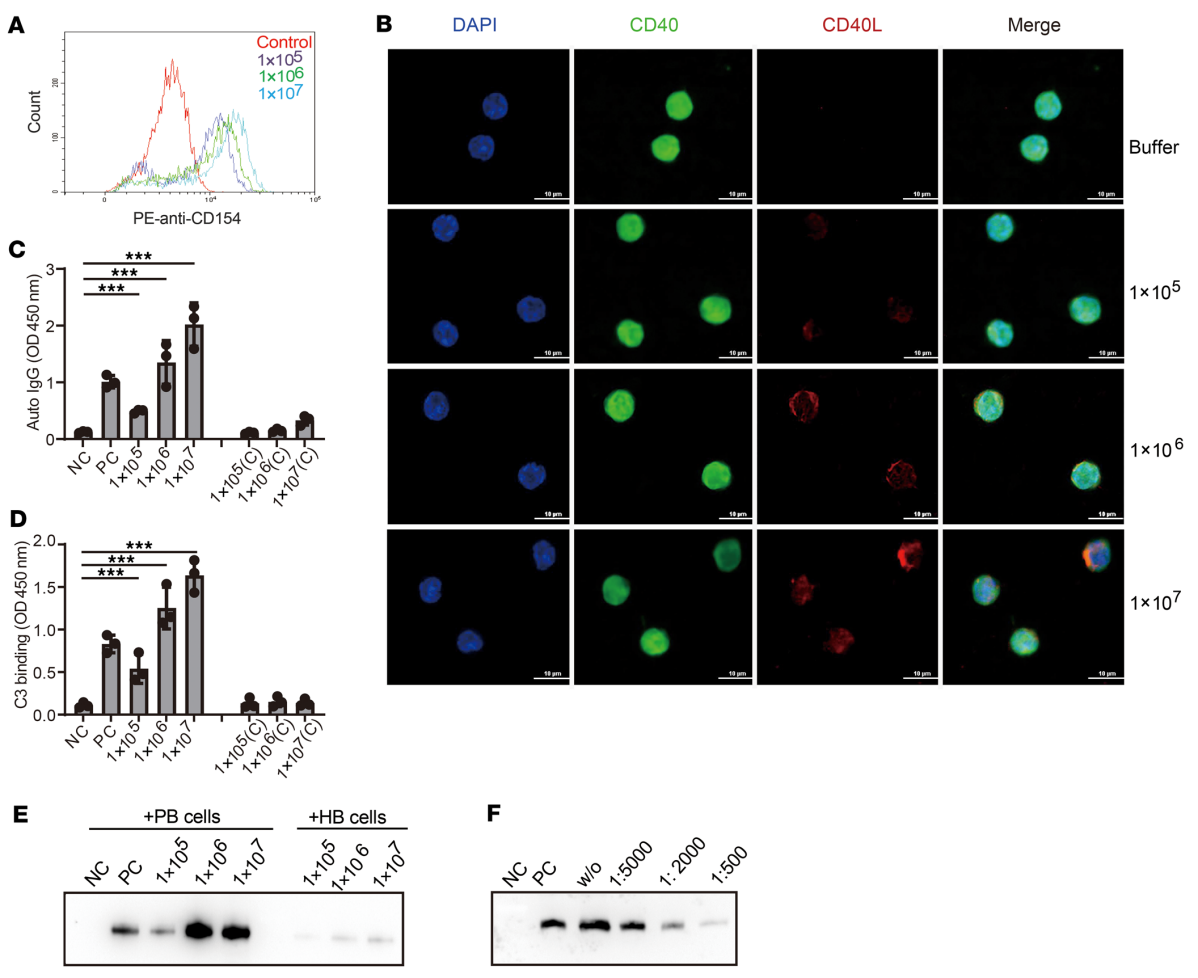
Pathogenic *C*. *albicans* infection–promoted autoantibody production was mediated by platelet-released CD40L. (**A**) Binding of CD40L to B cells analyzed by flow cytometry. (**B**) Co-localization CD40L and CD40 on the surface of B cells. Memory B cells isolated from P1 (1 × 10^6^/sample) were incubated with the platelet–*C*. *albicans* culture supernatant. Co-localization of CD40L and CD40 was examined by confocal microscopy. Representative images (out of 3) are shown. Scale bar: 10 μm. (**C**) Combined autoantibody release detected by ELISA. (**D**) Specificity of released autoantibody against C3 analyzed by ELISA. (**E**) Specificity of released autoantibody against FXIII analyzed by Far Western blot. (**F**) Neutralization of CD40L blocked autoantibody release by memory B cells. (**C**–**F**) CD40L-containing supernatant was added to the isolated memory B cells in the presence or absence of anti-CD40L. Purified CD40L was used as a positive control. Data are shown as mean ± SD; 1-way ANOVA was used for statistical analysis. ***P < 0.001. NC, negative control; PC, positive control; HB, memory B cells isolated from healthy donors; PB, memory B cells isolated from patient 1.

Afterward, to analyze whether platelet-released CD40L could bind to B cells, the supernatant from the platelet–*Candida* co-culture was incubated with peripheral memory B cells isolated from P1. Subsequently, the bound CD40L was quantified using flow cytometry. The results revealed that released CD40L dose-dependently bound to B cells, meaning that as the CD40L levels increased in the supernatant, the binding became stronger (Figure 8A). CD40L is a known ligand of the CD40 molecule (19). Consequently, the co-localization of CD40L and CD40 on the B cell surface was examined using confocal microscopy, and we found that CD40L bound and co-localized with CD40 on the B cell surface. The more CD40L present in the supernatant, the stronger the signal was, whereas CD40 was equally distributed on the B cell surface (Figure 8B).

Furthermore, we investigated whether the co-localization of CD40L with CD40 induces autoantibody production. Briefly, the co-culture supernatant containing different concentrations of CD40L was added and incubated with memory B cells previously isolated from P1 or the healthy donors. Five days after incubation, the released IgG was detected using ELISA. The results revealed that platelet-related CD40L promoted the production of autoantibodies from memory B cells. Conversely, even with the highest dose of *C*. *albicans* co-culture supernatant, almost no autoantibodies were detected in B cells isolated from the healthy control (Figure 8C). Subsequently, the specificity of these generated auto-IgG against C3 and FXIII was further confirmed by sandwich ELISA and Far Western blot (Figure 8, D and E).

To further validate the role of CD40L in mediating memory B cell activation, different dilutions of anti–CD40L antibody were applied to the aforementioned co-culture experiment. After co-incubation, the released autoantibodies were again detected using Far Western blot. The results suggested that the anti–CD40L antibody used at a 1:5,000 dilution slightly inhibited the release of autoantibodies. Addition of more anti–CD40L antibodies (1:2,000) caused almost 50% inhibition, and anti-CD40L used at a 1:500 dose almost completely inhibited the autoantibody release by memory B cells (Figure 8F). These data indicated platelet-released CD40L, triggered by fungal stimulation, bound to and stimulated memory B cells to produce autoantibodies, most likely through ligation of the surface-expressed CD40.

**Figure 9:**
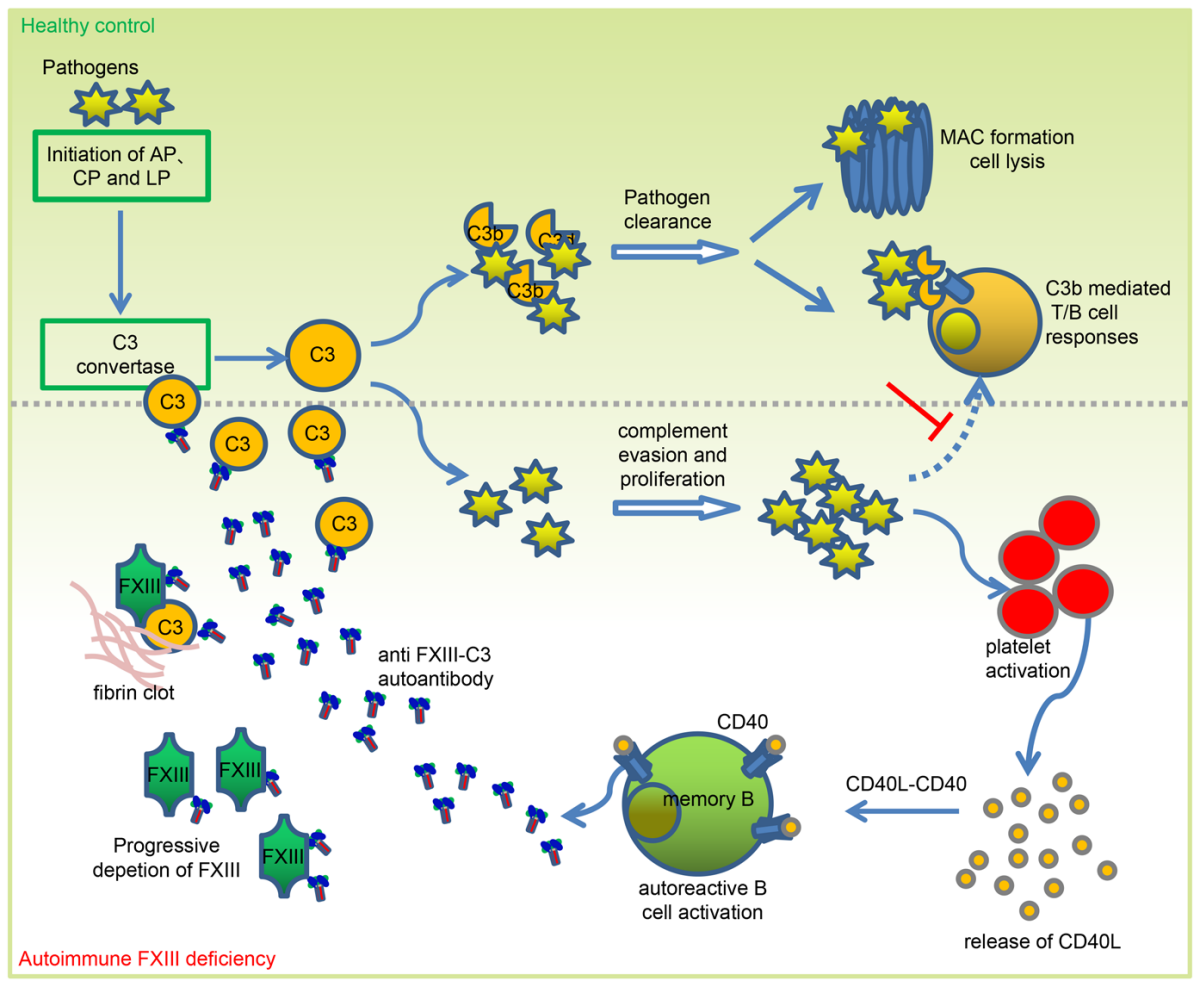
A model of action about the self-enhanced amplification loop of combined FXIII-C3 autoantibody generation for autoimmune FXIII deficiency. A combined FXIII-C3 autoantibody was identified in patients with autoimmune FXIII deficiency. The combined autoantibody, by forming a complex with C3, blocks C3 cleavage by C3 convertase, thereby inhibiting complement activation and complement mediated T cell activation, which increases host susceptibility to pathogenic *C*. *albicans* infections. Uncontrolled exogenous infections, by triggering platelets’ activation and platelet-related CD40L release, further promote autoantibody generation, resulting in progressive consumption of FXIII and C3. This process likely forms a self-enhanced amplification loop for autoantibody generation and progressive consumption of FXIII and C3 antigens.

Collectively, combined FXIII-C3 autoantibodies are present in patients with autoimmune FXIII deficiency. By neutralizing FXIII, these combined autoantibodies cause bleeding disorders. By forming a complex with C3, these autoantibodies block C3 cleavage by C3 convertase and further mediate fast clearance of C3 from blood clearance, thereby inhibiting complement activation and complement-mediated T cell activation, which increases host susceptibility to *C*. *albicans* infection. Uncontrolled *Candida* infections further trigger platelet activation and platelet-related CD40L release. By binding to CD40 on the memory B cell surface, released CD40L further induces combined FXIII-C3 autoantibody production, resulting in progressive deletion or clearance of FXIII and C3. Ultimately, a self-amplification loop for autoantibody generation is generated (Figure 9).

## Discussion

Autoimmune FXIII deficiency is a rare hemorrhagic disease characterized by severe recurrent spontaneous bleeding and high mortality rates (20). Our clinical investigation, together with in vivo mouse experiments, demonstrated that autoantibody generation in patients with autoimmune FXIII deficiency was positively correlated with pathogenic infection. Attributed to combined autoantibody-mediated complement inactivation, exogenous *C*. *albicans* invades patients and amplifies autoimmune signals to produce more autoantibodies. This phenomenon is consistent with a recent report suggesting that patients with preexisting autoimmune diseases are likely to experience amplified autoimmune responses when encountering secondary infections due to immune dysfunction (11).

In this study, a combined autoantibody against FXIII-C3 was identified in patients with autoimmune FXIII deficiency, which binds both FXIII and C3. Nikolajsen et al. (21) reported that during the transglutaminase-catalyzed reaction, the subunit A of FXIII-C3 was covalently linked within the fibrin clots. This linkage is similar to the binding of α2-antiplasmin to the coagulation site. This process induces an immune response and clears pathogens and damaged cells, thereby protecting the wound from infection (21). This likely indicates the existence of FXIII-C3 autoantigen in the body and explains why combined FXIII-C3 autoantibodies are produced in patients with autoimmune FXIII deficiency. To prove the existence of FXIII-C3 autoantigen, and further obtain an appropriately oriented FXIII-C3 autoantigen, we tried a phage display approach together with ELISA and Far Western blot. The data showed that binding of FXIII to C3 was detected only when the thrombin, as well as Ca^2+^, was present in the solution (Supplemental Figure 3A), indicating the natural structure of FXIII may conceal its binding site to C3 and, in the presence of Ca^2+^, thrombin may activate FXIII to better expose its linear epitope for C3 binding. These data are consistent with the report by Nikolajsen et al. (21). The Far Western blot data further proved that C3 bound to linear FXIII; however, FXIII did not bind to linear or denatured C3 (Supplemental Figure 3B). Likely, the natural spatial conformation of C3, but not the unfolded linear epitope, is required for FXIII binding. From this point of view, FXIII-C3 autoantigen likely retains the natural structure and function of C3. These data may also explain the phenomenon reported by Nikolajsen et al. (21) that cross-linking complement C3 within the fibrin clot induces an immune response and clears pathogens, thereby protecting the wound from infection.

In addition to bleeding disorders, patients with autoimmune FXIII deficiency exhibit decreased complement activation. In vitro data demonstrated that the combined FXIII-C3 autoantibody identified in these patients formed a complex with C3, thereby inhibiting C3 cleavage by preformed C3 convertase. These inhibitory effects were also observed for the C3-inhibitory domain of Efb (Efb-C) from the Gram-positive bacterium *Staphylococcus* *aureus*. Hammel et al. (22) performed structural analysis and found that Efb-C forms a complex with C3, thereby changing its spatial conformation and blocking its cleavage by the C3 convertase and further downstream activation of the complement responses. However, whether the binding of the combined FXIII-C3 autoantibody to C3 also triggers C3 spatial conformational change requires further exploration.

Complement is evolutionary in nature and forms a close network with coagulation and fibrinolytic systems. It participates in tightly regulated processes and is crucial for maintaining immune homeostasis in the body (23, 24). Upon infection, the complement system is activated within seconds to eliminate pathogens through MAC-mediated direct cytolytic effects and C3d-mediated antipathogenic T and B cell immune responses (25). Combined FXIII-C3 autoantibodies identified in patients with autoimmune FXIII deficiency interfere with complement activation and complement-mediated adaptive immune responses because of the lack of an effective first activation signal from complement activation fragments (26), which, in turn, increases host susceptibility to *C*. *albicans* infection, as observed in in vivo mouse models. As for the clinical patients, 4 of 5 patients with autoimmune FXIII deficiency experienced infections right before or when diagnosed. Although the fourth patient (P4) was not infected when diagnosed, this patient reported they were prone to catching a cold regularly. Additionally, in vivo mouse experiments confirmed that mice with autoimmune FXIII-C3 deficiency were more susceptible to *C*. *albicans* infections than were WT mice, as observed by higher fungal burden, severe pathological changes in kidneys, and shorter survival rate, which are common parameters to quantify host susceptibility to pathogenic infections in vivo (27–29).

Many hemolytic and thrombotic diseases are associated with complement dysfunction, including paroxysmal nocturnal hemoglobinuria, AIHA, aHUS, and DIC (30–32). Chen et al. (17) identified a combined autoantibody against C3b and factor B in patients with aHUS. In contrast to the combined FXIII-C3 autoantibody, the combined C3b-Bb autoantibody overactivated the complement system by stabilizing C3 convertase, which ultimately damaged the endothelial cells of the kidneys. Additionally, autoantibody-mediated complement activation on platelets was identified as a major mechanism of platelet destruction and removal in patients with immune thrombocytopenic purpura (33).

Lichter et al. (13) reported that greater than 74% of patients with SARS-CoV-2 and hospitalized in an ICU had defective FXIII levels and functions, and that FXIII levels gradually declined during infection with frequent severe bleeding, indicating that infection likely triggers progressive consumption of FXIII. Consistent with this phenomenon, we found that among the 5 patients with autoimmune FXIII deficiency, P1, P2, P3, and P5, who had infection, had lower FXIII levels or activity and higher combined autoantibody levels than P4, who did not have infection. However, the small number of patients remains a practical issue because of the rarity of such an autoimmune FXIII deficiency disease (since 2019, only 11 samples have been collected.). Therefore, to further confirm this phenomenon, mouse models of autoimmune FXIII-C3 deficiency were generated by intramuscularly injecting C3-FXIII fusion protein into mice to stimulate the production of combined FXIII-C3 autoantibodies, likely mimicking the conditions of patients with autoimmune FXIII deficiency.

Using autoimmune FXIII-C3-deficient mouse models, we found that *C*. *albicans* evaded host immune surveillance and proliferated in mice because of defective complement activation and down-stream antifungal T cell responses. Uncontrolled exogenous fungal infection further induced the combined autoantibody production, as evidenced by the accumulation of combined autoantibodies, indicating that *C*. *albicans* infection could also trigger autoimmune responses. Liu et al. (7) have pointed out that infection is an important environmental factor that induces the development of autoimmune diseases. Pathogens can typically induce instantaneous autoantibody production in the host within the first week after infection, and the quantity of autoantibodies produced is closely correlated with the host’s susceptibility (7, 8). However, when hosts (patients) already have an autoimmune deficiency, autoimmune signals are likely amplified by infections, resulting in a substantially higher number of autoantibodies (11), which explains why autoimmune FXIII-deficient mice produce much higher levels of autoantibodies when infected.

In terms of mechanism, uncontrolled *C*. *albicans* infection in autoimmune FXIII-C3-deficient mice triggered platelet activation and subsequently caused platelet-related CD40L release. These released CD40L, by binding to CD40, further activated and promoted autoreactive B cells to produce more autoantibodies. Wang et al. (10) reported that HIV bound to platelets through the Tat protein, thereby activating platelets to release CD40L molecules, which subsequently promoted autoreactive B cell activation, leading to the development of HIV-1–related autoimmune thrombocytopenia. Moreover, Meabed et al. (19) reported that platelet-released CD40L in the serum promoted B cell proliferation, differentiation, allotype conversion, and memory B cell generation via interaction with the B cell surface molecule CD40. These findings align with our results that CD40L secreted by activated platelets is crucial for inducing memory B cell activation by binding to CD40. Therefore, targeting CD40 on the B cell surface or neutralizing CD40L released by platelets could be promising targets to disrupt the self-amplification loop of autoantibody generation, with implications for potential therapeutic strategies to treat autoimmune FXIII deficiency, which will be the focus of our future research.

Overall, this clinical investigation, coupled with in vivo mouse model experiments and in vitro molecular analysis, identified combined FXIII-C3 autoantibodies in patients with autoimmune FXIII deficiency. These autoantibodies are positively correlated with pathogenic infection. Attributed to combined autoantibody-mediated complement inactivation, uncontrolled *C*. *albicans* invades patients and further triggers platelet activation and platelet-related CD40L release, thereby promoting memory B cell activation to produce more autoantibodies by binding to CD40 on the B cell surface. This process likely forms a self-amplification loop for the progressive depletion of FXIII and C3. We believe this study offers insights and therapeutic guidance for the improved management and treatment of autoimmune FXIII deficiency.

## Methods

### Sex as a biological variable.

In our study, both female and male mice were used, and similar findings are reported for both sexes. Therefore, sex was not considered a biological variable.

### Antibodies, proteins, ELISA kits, serum, and plasma.

HRP-labeled donkey anti–goat (Solarbio, K1038D-HRP), goat anti–rabbit (Abcam, ab6721), chicken anti–human IgG (Abcam, ab112453), and goat anti–mouse IgG (Proteintech, SA00001-1) were used for WB analysis as secondary antibodies. All fluorescently labeled antibodies used for flow cytometry analysis and immunofluorescent staining are listed in Supplemental Table 2. Human C3 (CompTech, A113), mouse C3 (AntibodySystem, AMB89901), human FXIII-A (Elabscience, PKSH033713), human FXIII-B (Elabscience, PDMH100391), and murine FXIII-A (MedChemExpress, 9013-56-3) were used for autoantibody detection. Human C5a ELISA kit (442107), mouse TNF-α (430907), INF-g (430807), and IL-17 ELISA kits (436207) were all purchased from BioLegend. The mouse C3a ELISA kit was bought from Elabscience (catalog E-EL-M0337). Human CD40L ELISA kit (catalog Ab99991) and mouse CD40L ELISA kit (catalog ab119517) were from Abcam.

Patient serum or plasma samples were collected from patients with clinically diagnosed FXIII deficiency, and NHS or NHP was collected from 5 healthy donors and pooled together. All sera were stored at –80°C until use.

### Autoantibody detection.

To detect anti–FXIII or anti–C3 autoantibody levels in plasma, purified FXIII (subunit A or B; 0.5 μg/well in 100 μL of PBS) or C3 was coated onto plates overnight at 4°C. After washing, the plate was blocked with 4% milk powder for 2 hours at room temperature (RT), followed by incubation with plasma (1:50 dilution) for 1 hour at 37°C. Bound antibodies were detected using HRP-labeled chicken anti–human or goat anti–mouse IgG antibodies (1:4,000).

To detect combined FXIII-C3 autoantibodies, FXIII-A was initially immobilized. After blocking, diluted plasma (1:50) was added to capture anti–FXIII autoantibodies. After washing, purified C3 was added and incubated at 37°C for 1 hour. Finally, bound C3 was detected using a specific anti–human C3 antibody (CompTech, A213).

### Autoantibody purification.

Sepharose 4B column (HiTrap NHS activated human plasma; 17-0716-01, GE Healthcare) precoupled with FXIII was washed thrice with 0.01 M Trisbase, followed by incubation with 0.5% BSA for 2 hours to block free sites. Subsequently, the plasma of P1 (10 mL) prediluted with 0.01 M Tris base was slowly loaded into the column at 4°C, followed by washing 3 times. The coupled anti–FXIII autoantibody was further eluted with 0.1 M glycine (pH 2.5). The eluted antibody was collected in tubes containing 1 M NaHCO_3_ for neutralization, followed by overnight dialysis in PBS. These purified antibodies were qualified using Coomassie staining and Far Western blot.

### Construction of mouse fusion protein C3-FXIII.

The constructed fusion protein C3-FXIII contains a mouse C3-α chain (Thr671-Asn1663), mouse FXIII-A (Met1-Met732), a linker (GGGGS)_3_ in the middle, and a C-terminal Strep-tag. Briefly, the gene sequence corresponding to the 2 subunits, as well as the linker, signal peptide, and sequences containing restriction sites (EcoRI/NotI), were directly synthesized and inserted into pATX2/To/myc-Strep. Recombinant C3-FXIII protein was expressed in HEK 293 cells (ACC305) by transient transfection with polyethylenimine (jetPEI; Polyplus, 101000020). Secreted proteins were purified from the culture supernatant using affinity chromatography, and the purity was proved using Coomassie staining.

### Complement activation assay.

Complement activation of 3 different pathways was performed as described previously (34). Briefly, LPS (10 μg/mL, for AP), mannan (100 μg/mL, for LP), or 2 μg/mL IgM (for CP) were precoated in 96-well plates. After washing with PBS, the wells were blocked with 4% milk powder for 2 hours at RT and rewashed with HBS^2+^ (20 mM HEPES, 140 mM NaCl, 5 mM CaCl_2_, and 2.5 mM MgCl_2_, pH 7.4, for CP and LP) or Mg-EGTA buffer (20 mM HEPES, 144 mM NaCl, 7 mM MgCl_2_, and 10 mM EGTA, pH 7.4, for AP). The serum of patient or healthy control donors was first diluted (1:50 for LP and CP, and 1:5 dilution for AP), then added to wells and incubated for 1 hour at 37°C. Finally, MAC formation and C3b/iC3b deposition were detected using polyclonal rabbit anti–human MAC (CompTech, A227) or polyclonal goat anti–human C3 antibodies, followed by HRP-labeled goat anti–rabbit or donkey anti–goat IgG as secondary antibodies.

To examine the effects of anti–FXIII autoantibodies on complement activation, 96-well plates were coated with LPS, followed by the blocking and washing steps described above. NHS, preincubated with FXIII-fished anti–FXIII autoantibodies from different patients or different amounts of purified anti–FXIII autoantibodies, was subsequently added to the plates and incubated for 1 hour at 37°C. Following washing, MAC formation and C3b/iC3b deposition were detected using specific antibodies, as described above. Isotype IgG and commercial anti–human FXIII were used as controls.

### Effects of anti–FXIII autoantibody on C3 convertase.

C3b-coated particles were initially prepared to analyze the effects of autoantibodies on C3 convertase formation (35, 36). Briefly, NHS (30%) was activated with zymosan (Bioss, D10315) (3 mg/mL) for 30 minutes in HBS^2+^ buffer. After washing, the particles were incubated in PBS for 30 minutes at 37°C to dissociate C3 convertases and obtain C3b-coated particles. Subsequently, factors B (CompTech, A135) (40 mg/mL), D (CompTech, A136) (1 mg/mL), and P (CompTech, A139) (4 mg/mL) or C3b-coated particles were preincubated with purified autoantibodies, isotype IgG, or commercial anti-FXIII as controls for 15 minutes at 37°C. The 2 parts were mixed and incubated for 30 minutes at 37°C. The C3bBb levels on the particle surface were analyzed by flow cytometry using an anti–human Bb monoclonal antibody (Quidel, A712) to quantify C3 convertase formation.

To analyze the effects of autoantibodies on C3 cleavage, C3 convertase was generated on the zymosan surface (35). Briefly, NHS (30%) was activated by zymosan (3 mg/mL) for 30 minutes in HBS^2+^ buffer. After washing, the particles were incubated in PBS for 30 minutes at 37°C to dissociate the C3 convertases. Subsequently, factors B (40 mg/mL), D (1 mg/mL), and P (4 mg/mL) were added and incubated for 30 minutes at 37°C. Meanwhile, C3 was preincubated with different amounts of purified anti–FXIII autoantibody, PBS, isotype IgG, or commercial anti-FXIII as controls in buffer supplied with 0.05 μg/mL BSA for 15 minutes at 37°C, then added to the preformed C3 convertase and further incubated for 30 minutes at 37°C. Finally, the C3a level in the supernatant was analyzed by WB. Because the incubation buffer contained 0.05 μg/mL BSA, BSA was used as a loading control in WB analysis, which was detected using a polyclonal rabbit anti–BSA antibody (Solarbio, SPA163), followed by HRP-labeled goat anti–rabbit as the secondary antibody.

### In vivo mouse experiments.

All mice (male and female equally distributed; 8–10 weeks old; 20–28 g) used for this study were on a C57BL/6 background. To mimic the autoimmune FXIII deficiency condition, C57BL/6 mice were intramuscularly injected with PBS, FXIII, C3, or fusion protein C3-FXIII (50 μg/mouse injection dose; *n* = 12 for each group of mice) at day –16 and refreshed at day –9 (37, 38), generating 4 groups of mice (simply named as WT, FXIII, C3, and FXIII-C3). Blood samples were collected on days –8, –6, and –2. Combined autoantibodies against FXIII-C3, monospecific antibodies against FXIII or C3, and FXIII and C3 levels were quantified to validate the successful establishment of the models.

The established mouse models were further used for in vivo analysis of host susceptibility to infection by *C*. *albicans* (SC5314, provided by Bernhard Hube, Department of Microbial Pathogenicity Mechanisms, Leibniz Institute for Natural Product Research and Infection Biology, Hans Knöll Institute, Jena, Germany). Briefly, mouse survival rates were monitored for 15 days after the intravenous injection of *C*. *albicans* (1 × 10^5^/mouse) into WT, FXIII, C3, and FXIII-C3 groups (*n* = 10). Meanwhile, the tail bleeding time was analyzed to determine the bleeding tendency, and the patterns of immune responses, fungal load, and kidney pathology in these groups of mice were examined after infection (39). Briefly, on day 0, *C*. *albicans* (5 × 10^4^/mouse) or PBS was intravenously injected into WT, FXIII, C3, and FXIII-C3 groups of mice, generating a total of 8 groups of mice classified as WT/Ca versus WT/PBS, FXIII/Ca versus FXIII/PBS, C3/Ca versus C3/PBS, and FXIII-C3/Ca versus FXIII-C3/PBS (*n* = 6/group). On days 1, 5, and 7 after infection, blood samples were collected to analyze the plasma levels of combined autoantibodies or monospecific autoantibodies against FXIII or C3. On day 7 after infection, tail bleeding time was first recorded, then the mice were sacrificed, blood was collected to analyze cytokine release, C3a release, FXIII levels, and CD40L, using an ELISA kit or WB. Platelet activation was analyzed via flow cytometry by quantifying CD62P expression. The spleen, brain, liver, lung, and kidneys were collected to quantify the fungal burden, and part of the kidneys were fixed for H&E staining and immunofluorescent staining to analyze kidney injury, C3b deposition, and T cell infiltration. H&E-stained slides were scanned by NanoZoomer S360 (Hamamatsu Photonics) and the immunofluorescent stained slides were scanned by Pannoramic MIDI (3DHistech) Additionally, T cells were isolated from the spleen, and the portion of CD44^high^/CD62L^low^ and IL-17^+^, and IFN-g positive CD4 or CD8 T cells was analyzed using flow cytometry (CytoFLEX, Beckman) to quantify T cell activation (40). Furthermore, B cell activation was quantified by the percentage of CD69^+^ B cells among CD19^+^ cells, and autoreactive B cell activation was quantified by the percentage of anti–FXIII IgG^+^–activated B cells. In total, 3 batches of mouse experiments were conducted.

### In vitro co-culture experiments.

To analyze whether co-incubation of *C*. *albicans* with platelets can activate platelets and induce CD40L release, different doses of *C*. *albicans* (1 × 10^5^, 1 × 10^6^, and 1 × 10^7^/sample) were incubated with platelet-rich plasma (1 × 10^7^/sample in 200 μL) in the presence of Gly-Pro-Arg-Pro (Pefa 6003) (MedChemExpress, HY-P0074) to prevent platelet aggregation for 60 minutes at 37°C (10, 41). Subsequently, platelet activation was assessed by quantifying the expression of surface CD62p and GPIIbIIIa using flow cytometry. CD40L release in the culture supernatant was analyzed using an ELISA kit. Subsequently, to analyze CD40L binding to CD40 on B cell surfaces, peripheral memory B cells were isolated from the blood of P1 using a memory B cell selection kit (Miltenyi, 130-093-546) and co-incubated with the supernatant of *C*. *albicans*–platelets co-culture for 30 minutes at 37°C. The bound CD40L on the B cell surface was quantified by flow cytometry using a FITC-labeled anti–human CD40L antibody (BioLegend, 310804). The co-localization of CD40L with CD40 on the B cell surface was examined using confocal microscopy (Nikon A1 plus, Nikon Corp.) after staining with APC-labeled anti–human CD40 (BioLegend, 334309; 1:200) and FITC-labeled anti–human CD40L (1:200).

To analyze CD40L-CD40 ligation-mediated antibody production, memory B cells were isolated from P1 or healthy donors (control groups). Subsequently, CD40L-containing supernatant, prepared by co-culturing platelets (1 × 10^6^) with *C*. *albicans* (1 × 10^5^, 1 × 10^6^, and 1 × 10^7^), was added to the isolated B cells and further cultured for another 5 days. Purified CD40L (Elabscience, PKSH032214) was used as a positive control. The released autoantibodies in the supernatant and specificity to FXIII and C3 were analyzed using ELISA or Far Western blot. To further validate the role of CD40L in mediating autoantibody release, different amounts of anti–human CD40L antibody (BioLegend, 591702) were applied to the aforementioned co-culture system. Again, the released anti–FXIII autoantibody was analyzed using Far Western blot.

### Statistics.

Statistical analyses were performed using the GraphPad Prism 9.0. One-way ANOVA or unpaired 2-tailed Student’s *t* test was used for statistical analysis, and the mouse survival rate was specifically analyzed using the Kaplan-Meier log-rank test. Data are expressed as the mean ± SD. Group sizes and reproducibility are indicated in the figure legends. All images shown are representative of a minimum of 3 independent experiments *P* values of less than 0.05 are considered significant (*P < 0.05, **P < 0.01, ***P < 0.001).

### Study approval.

This study was approved by the Ethical Board of the Tongji Medical College, Huazhong University of Science and Technology, China (ethics code: UHCT-IEC-SOP-016-03-01). Written consent was obtained from all patients for participation in the study. Serum and plasma samples were collected from 11 patients with FXIII deficiency and from 5 healthy donors.

Six-week-old C57BL/6 mice were housed under specific pathogen–free conditions at the Animal Care Center of Tongji Medical College, Huazhong University of Science and Technology (Wuhan, China) for the animal experiments. This study was conducted in accordance with the guidelines of the NIH for the Care and Use of Laboratory Animals. All mice study protocols were approved by the Institutional Animal Care and Use Committee of Tongji Medical College (permit 2695).

### Data availability.

All data that support the findings of this study are available in the main text or the supplemental materials. See the Supporting Data Values file for values underlying the data presented in each graph. The additional materials and methods information and the methods for ELISA, WB, Far Western blot, measurement of FXIII antigen level and activity, and the phage display approach are presented in the supplemental information.

## Author contributions

SL, DH, and YH designed the study and wrote the manuscript. SL performed the experiments, analyzed the data, and contributed to preparing figures. JD and Y Liu performed experiments, wrote part of the manuscript, and contributed to preparing figures and tables. LX, CW, WW, YD, BF, and YC performed partial in vivo mouse experiments and contributed to preparing tables. LZ, MZ, JF, HW, ZC, and YW provided clinical samples and conceptual advice. GZ, RZ, HQJ, Y Li, KH, and XC provided technical support. LVT, CS, HM, and PFZ provided reagents and conceptual advice. All authors reviewed the manuscript. SL, JD, and Y Liu share first authorship; the order was determined by their contributions. SL, HW, YW, DH, and YH are co-corresponding authors.

## Funding support

- National Key R&D Program of China (grant 2022YFC2304600).
- The National Natural Science Foundations of China (grants 82070136, 82274317, and 81974249).
- The Noncommunicable Chronic Diseases-National Science and Technology Major Project (grant 2023ZD0509900).
- The National Key Research and Development plan (grant 2023YFC2307003).
- Natural Science Foundation of Hubei Province (Nr. 2025AFD485)

## Supplementary Material

Supplemental data

Unedited blot and gel images

Supporting data values

## Figures and Tables

**Table 1:**
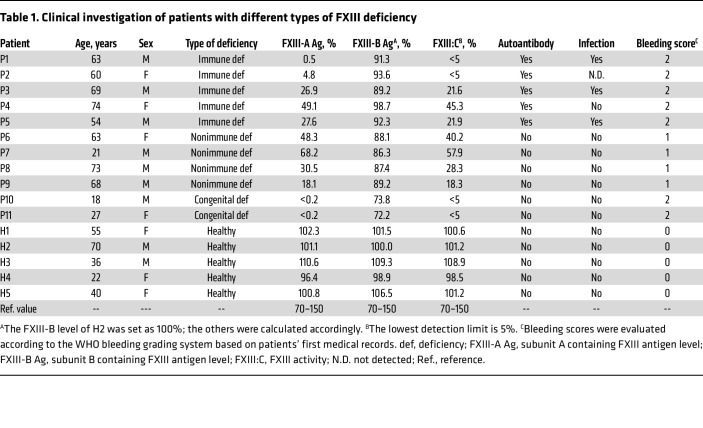
Clinical investigation of patients with different types of FXIII deficiency

## Version 1 12/09/2025

In-Press Preview

## Version 2 02/02/2026

Electronic publication
