# Supplementary material for: Combined FXIII-C3 autoantibodies elicit bleeding and complement dysfunction in autoimmune FXIII deficiency
Source: J Clin Invest. 2025 Dec 9;136(3):e192619. doi: 10.1172/JCI192619 (PMC12867163; doi:10.1172/JCI192619)
Supplement: Supplemental data [file jci-136-192619-s081.pdf]

Supplemental information

Combined FXIII-C3 autoantibodies elicit bleeding and complement dysfunction in autoimmune FXIII deficiency

Shanshan Luo[1#*], Jun deng[1#], Yue Liu[2#] , Lv Xiong[1], Wanting Wang[1], Chaofan Wang[1], Yaohua Cai[1], Yajie Ding[1], Bahgat Fayed[3], Zhipeng Cheng[1], Lu Zhang[1], Min Zhang[1], Jun Fang[1], Gensheng Zhang[4], Rui Zhu[5], Haiqiang Jiang[6], Yunlun Li[6], Kun Huang[7], Xiang Cheng[8,9], Liang V. Tang[1], Chunyan Sun[1], Heng Mei[1], Peter F Zipfel[10], Huafang Wang[1*], Yadan Wang[1*], Desheng Hu[5,9*], Yu Hu[1,11*]

[1]Institute of Hematology, Union Hospital, Tongji Medical College, Huazhong University of Science and Technology, Wuhan, China.

[2]National Clinical Research Center for Chinese Medicine Cardiology, Xiyuan Hospital, Chinese Academy of Chinese Medical Sciences, Beijing, China.

[3]Department of Chemistry of Natural and Microbial Products, Pharmaceutical and Drug Industries research institute, National Research Centre, Giza, Egypt.

[4]Department of Critical Care Medicine, Second Affiliated Hospital, Zhejiang University School of Medicine, Hangzhou, Zhejiang, China.

[5]Department of Integrated Traditional Chinese and Western Medicine, Union Hospital, Tongji Medical College, Huazhong University of Science and Technology, Wuhan, China.

[6]Innovation Research Institute of Traditional Chinese Medicine, Shandong University of Traditional Chinese Medicine, Jinan, China

[7]School of Pharmacy, Tongji Medical College, Huazhong University of Science and Technology, Wuhan, China

[8]Department of Cardiology, Union Hospital, Tongji Medical College, Huazhong University of Science and Technology, Wuhan, China.

[9]Hubei Key Laboratory of Biological Targeted Therapy, Union Hospital, Tongji Medical College, Huazhong University of Science and Technology, Wuhan, China.

[10]Department of Infection Biology, Leibniz Institute for Natural Product Research and Infection Biology, Hans Knöll Institute, Jena, Germany; Friedrich Schiller University, Faculty of Biological Sciences, Jena, Germany.

[11]Key lab of Molecular Biological Targeted Therapies of the Ministry of Education, Union Hospita, Tongji Medical College, Huazhong University of Science and Technology, Wuhan, China.

Conflict-of-interest: The authors have declared that no conflict of interest exists.

[#] These authors contribute equally to this work

Address correspondence to: Yu Hu, Institute of Hematology, Union Hospital, Tongji Medical College, Huazhong University of Science and Technology, 1277 Jiefang Avnue, Wuhan 430022, China. E-mail: dr_huyu@126.com, 008613986183871. or to Desheng Hu, Department of Integrated Traditional Chinese and Western Medicine, Union Hospital, Tongji Medical College, Huazhong University of Science and Technology, 1277 Jiefang Avnue, Wuhan 430022, China. E-mail: desheng.hu@hust.edu.cn, 008619023885858;

Supplemental Table 1: Prothrombin time (PT), activated partial thromboplastin time (APTT), thrombin time (TT), as well as qualitative analysis of FXIII activity were performed. The data showed that all these patients had normal prothrombin time, activated partial thromboplastin time and thrombin time. P1-P3 and P5 were positive in Urea dissolution assay, meaning that blood clot got lysed within two hours when mixed with Urea. This method is only qualitative analysis, but not very sensitive.

Supplemental Table 1

Patient Nr.	Urea dissolution assay	PT (sec)	APTT (sec)	TT(sec)
P1	+	12.9	44.3	14.6
P2	+	14.3	43.2	18.2
P3	+	13.6	36.6	15.3
P4	-	11.8	37.1	14.7
P5	+	15.6	39.1	16.2
P6	-	13.0	32.4	16.0
P7	-	12.2	40.5	15.1
P8	-	13.5	39.2	15.5
P9	+	15.6	31.2	20.7
P10	+	12.7	30.7	14.3
P11	+	12.0	34.8	16.1
Ref. value	not related	10.0--16.9	20.0--47.1	12.9-22.9

"+" meaning clot got lysed; "-" indicating clot was not lysed. This Urea dissolution assay is not sensitive. Normally, when the FXIII-A level is more than 30%, we hardly observe the clot lysis.

Supplemental Table 2. List of antibodies for flow cytometry analysis and immunofluorescent staining

Antibodies against	Flourescent label	Company	Catalog number
Mouse CD4	PEcy7	Biolegend	100528
Mouse CD8	Percp-cy5.5	BD	551162
Mouse CD44	BV421	Biolegend	103039
Mouse CD62L	APC-cy7	Biolegend	104428
Mouse IFNg	BV421	Biolegend	505830
Mouse IL-17A	Percp-cy5.5	BD	560666
Mouse CD19	PE	Biolegend	115508
Mouse CD69	FITC	Biolegend	104505
Mouse CD41	FITC	Biolegend	133903
Mouse CD62p	APC	Biolegend	148304
Human GPIIbIIIa	PE	Biolegend	305157
Human 62p	APC/Cyanine7	Biolegend	304944
Human CD40L	FITC	Biolegend	310804
Human CD40	APC	Biolegend	334309

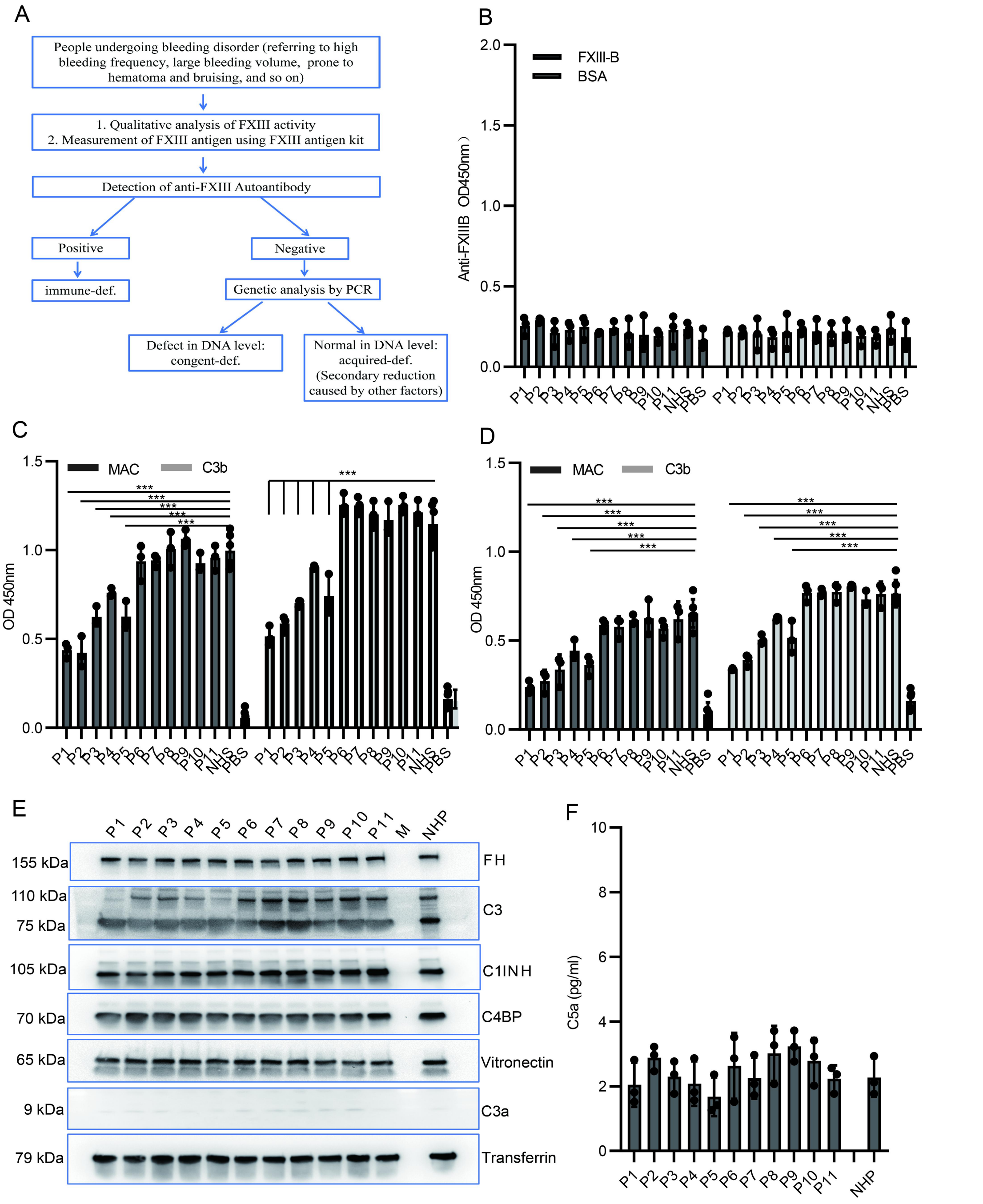

Supplemental Figure 1. Autoimmune FXIII deficient patients displayed deceased complement activation. **(A)** Flow char for identification and classification of FXIII deficient patients. **(B)** Detection of anti FXIII-B autoantobody. NHP, normal human plasma. **(C and D)** Anti-FXIII autoantibody blocks lectin pathway (LP, C) and classical pathway (CP, D) complement activation. For complement activation assays:100ug/ml Mannan (for LP) or 2ug/ml IgM (for CP) were pre-coated in 96 well

plate. After washing, the wells were blocked with 4% milk powder for 2h at room temperature. Then, the plate was further washed with HBS^{2+} buffer, serum from different patients or healthy donors were first diluted in HBS^{2+} (1:50), then added and incubated for 1h at 37°C. Following washing, MAC formation as well as C3b/iC3b deposition was detected with polyclonal rabbit anti-MAC antibody (1:3000) or polyclonoal goat anti-C3 antibody (1:4000) followed by HRP-labeled goat anti rabbit or rabbit anti-goat as secondary antibody (1:4000). NHS, normal human serum. (**E**) Detection of complement C3, C3a and complement inhibitors in patients and healthy controls by Western blotting. 11 patients' plasma as well as the pool of five normal human plasma (NHP) were treated under reducing conditions, separated by SDS-PAGE, then transferred into the membrane. After blocking, membranes were incubated with different antibodies specific for Factor H (FH), C3, C1 inhibitor (C1INH), C4b binding protein (C4BP), vitronectin and C3a. Transferrin was used as an internal reference. (**F**) Analysis of complement C5a level in plasma of 11 patients and healthy controls by ELISA. Representative blots out of three are shown. Data are shown as means ± SD. One way ANOVA was used for statistical analysis. *$p<0.05$, **$p<0.01$,***$p<0.001$.

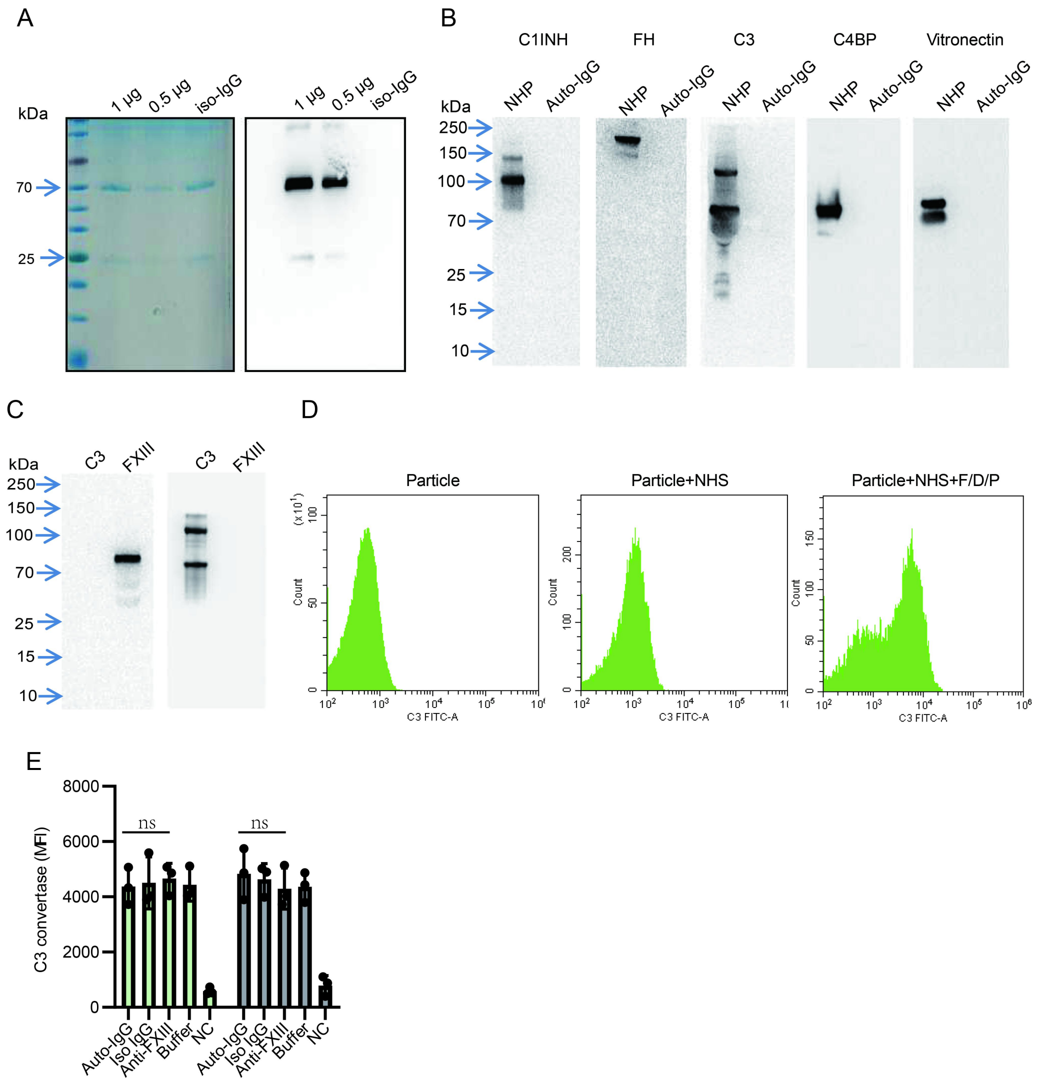

Supplemental Figure 2. A combined FXIII-C3 autoantibody was identified in autoimmune FXIII deficient patients, which by neutralizing C3, inhibited complement activation. (A) Quality verification of purified autoantibody from P1. Anti-FXIII autoantibody was purified from plasma of P1 using FXIII coupled affinity chromotography, and further verified by Coomassie staining and "Far Western" blot. Isotype human IgG (Iso-IgG) was used as controls. (**B**) Detection of complement inhibitors and C3 in purified anti-FXIII-C3 autoantibody (Auto-IgG). Normal human plasma (NHP) and purified Auto-IgG were treated under reducing conditions, separated by SDS-PAGE, then transferred to membrane. After blocking, membranes

were incubated with different antibodies specific for C1 inhibitor (C1INH), Factor H (FH), C3, C4b binding protein (C4BP) and vitronectin. NHP was used as positive controls. (**C**) Commercial anti-human FXIII and anti-human C3 do no cross-react with C3 or FXIII. C3 and FXIII were treated under reducing conditions, separated by SDS-PAGE, then transferred to membrane. After blocking, membranes were incubated with either commercial anti-human FXIII (left) or commercial anti-human C3 antibody (right). (**D**) Level of C3 convertase on zymosan surface. C3 convertase was build up on a zymosan surface using purified complement proteins, the level of C3 covertase formed on the surface was quantified by flow cytometry using specific anti-human Bb antibody. **(E)** Effects of anti-FXIII autoantibody (Auto-IgG) on C3 convertase formation. Individual complement proteins, Factor B (FB), Factor D (FD) and Factor P (FP), or surface attached C3b were pre-incubated with Auto-IgG, Iso-IgG, Commercial anti-FXIII or buffer, then combined together for further incubation. The C3 convertase level were then quantified by flow cytometry. Median fluorescent intensity (MFI) was used for further analysis. Data are shown as means ± SD. Representative histogram and blots out of three was shown. Unpaired two tailed Student's t-test was used for statistical analysis *$p<0.05$, **$p<0.01$,***$p<0.001$.

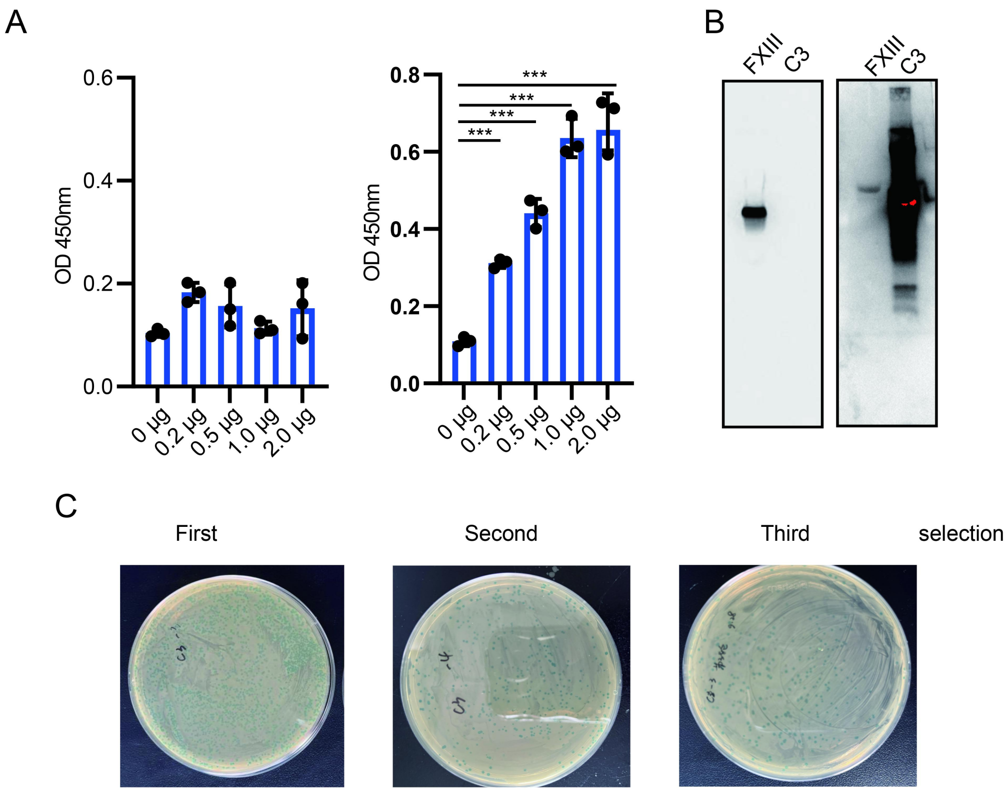

D

C3 coated						BSA control					
0.0082	0.046	0.0554	0.0042	0.0019	0.0056	0.0888	0.0271	0.0436	0.0109	0.0701	0.0382
0.0003	0.0457	0.0009	0.0017	0.0082	0.0456	0.0155	0.0196	0.035	0.0546	0.0892	0.0362
0.0402	0.0458	0.4491	0.0041	0.0158	0.0174	0.0455	0.0448	0.0318	0.0577	0.0839	0.0005
0.048	0.247	0.002	0.0445	0.0063	0.0089	0.028	0.0281	0.0277	0.0482	0.0012	0.0486
0.0024	0.0088	0.0087	0.0111	0.0166	0.0097	0.0362	0.0176	0.043	0.0903	0.0401	0.0494
0.0004	0.0028	0.0296	0.0438	0.0236	0.0148	0.0216	0.0132	0.004	0.0994	0.0055	0.0121
0.0442	0.0456	0.0359	0.0358	0.014	0.0418	0.0937	0.0435	0.0654	0.0321	0.093	0.095
0.0879	0.0498	0.0159	0.0071	0.0046	0.0668	0.0242	0.0633	0.0593	0.0198	0.0144	0.0671

E

```
>NP_000120.2 coagulation factor XIII A chain [Homo sapiens]
MSETSRTAFGGRRAVPPNNSNAAEDDLPTVELQGVVPRGVNLQEFLNVTSVHLFKERWDTNKVDHHTDK
YENNKLIVRRGQSFYVQIDFSRPYDPRRDLFRVEYVIGRYPQENKGTYIPVPIVSELQSGKWGAKIVMR
EDRSVRLSIQSSPKCIVGKFRMYVAVWTPYGVLRTSRNPETDTYILFNPWCEDDAVYLDNEKEREEYVL
NDIGVIFYGEVNDIKTRSWSYGQFEDGILDTCLYVMDRAQMDLSGRGNPIKVSRVGSAMVNAKDDEGVL
VGSWDNIYAYGVPPSAWTGSVDILLEYRSSENPVRYGQCWVFAGVFNTFLRCLGIPARIVTNYFSAHDN
DANLQMDIFLEEDGNVNSKLTKDSVWNYHCWNEAWMTRPDLPVGFGGWQAVDSTPQENSDGMYRCGPAS
VQAIKHGHVCFQFDAPFVFAEVNSDLIYITAKKDGTHVVENVDATHIGKLIVTKQIGGDGMMDITDTYK
FQEGQEEERLALETALMYGAKKPLNTEGVMKSRSNVDMDFEVENAVLGKDFKLSITFRNNSHNRYTITA
YLSANITFYTGVPKAEFKKETFDVTLEPLSFKKEAVLIQAGEYMGQLLEQASLHFFVTARINETRDVLA
KQKSTVLTIPEIIIKVRGTQVVGSDMTVTVEFTNPLKETLRNVWVHLDGPGVTRPMKKMFREIRPNSTV
QWEEVCRPWVSGHRKLIASMSSDSLRHVYGELDVQIQRRPSM
```

Supplemental Figure 3. Identification of the C3 binding sequence within FXIII-A by screening a phage array. (A) In vitro analysis of FXIII-C3 interaction by ELISA. (**B**) Linear binding of FXIII with C3 analyzed by "far western" assays. FXIII and C3 were treated under reducing conditions, then subjected to the SDS-PAGE, transferred

to PVDF membrane. Following blocking, membrane were incubated with C3 (left) or FXIII-A (right), then detected by anti human C3 (left) or anti human FXIII (right) antibodies. (**C**) Positive clones after three round selections of the phage display. (**D**) Binding of selected clones with C3 analyzed by ELISA. Randomly selected 48 clones from the plates after the third selection, further analyzed their binding ability to coated C3 (left panel), BSA was used as negative controls (right panel). (**e**) Two possible binding epitopes located within the N-terminus of FXIII-A. Two positive clones were selected, sequenced as FTAPYPR and LTPFVHL. When further aligned with FXIII protein sequence, these two possible binding epitopes are located within the N-terminal of FXIII-A with ca. 70% match, highlighted in red. Representative blots out of three are shown. Data are shown as means ± SD. One way ANOVA was used for statistical analysis. *p<0.05, **p<0.01,***p<0.001.

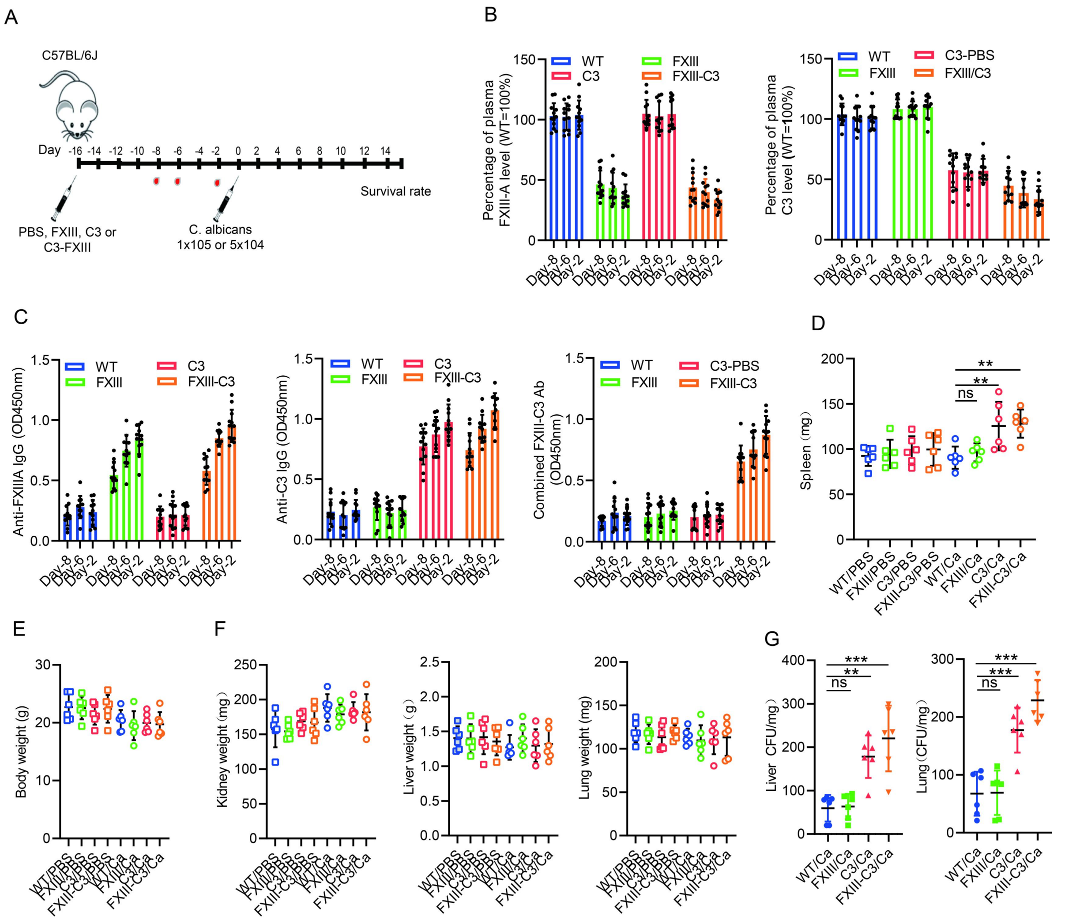

Supplemental Figure 4. Autoimmune FXIII deficient mice suffered severe pathogenic *C. albicans* infections. (A) The time schedule of in vivo mice experiments. C57BL/6J mice were first immunized by PBS, FXIII, C3 or C3-FXIII, generating four groups of mice, simply named WT, FXIII, C3, and FXIII-C3. (**B**) Pre-quantification of FXIII-A level and C3 in individual mouse at day -8, -6 and -2 upon PBS, FXIII, C3 or FXIII/C3 immunization (n=12 per group). FXIII-A antigen level (left) was detected using an FXIII antigen kit, complement C3 level (right) was analyzed by C3 ELISA kit. FXIII-A antigen level or C3 level of WT group at day -8 was set as 100%, the other samples were calculated accordingly. (**C**) Mono-specific antibodies against FXIII or C3, and combined FXIII-C3 autoantibody were measured

by "sandwitch" ELISA. (**D**) Spleen weight of individual mice. (**E**) Body weight of eight groups of mice upon infection. (**F**) Kidney, lung and liver weight of individual mice. (G) Fungal load of liver and lung. In the in vivo infection model, WT, FXIII, C3 and FXIII-C3 groups of mice were intravenously injected with *C. albicans* ($5x10^4$/mouse) or PBS, eight groups of mice were clarified as WT/Ca and WT/PBS, FXIII/Ca and FXIII/PBS, C3/Ca and C3/PBS, FXIII-C3/Ca and FXIII-C3/PBS (n=6 per group). Data are shown as means ± SD. One way ANOVA was used for statistical analysis. *p<0.05, **p<0.01,***p<0.001.

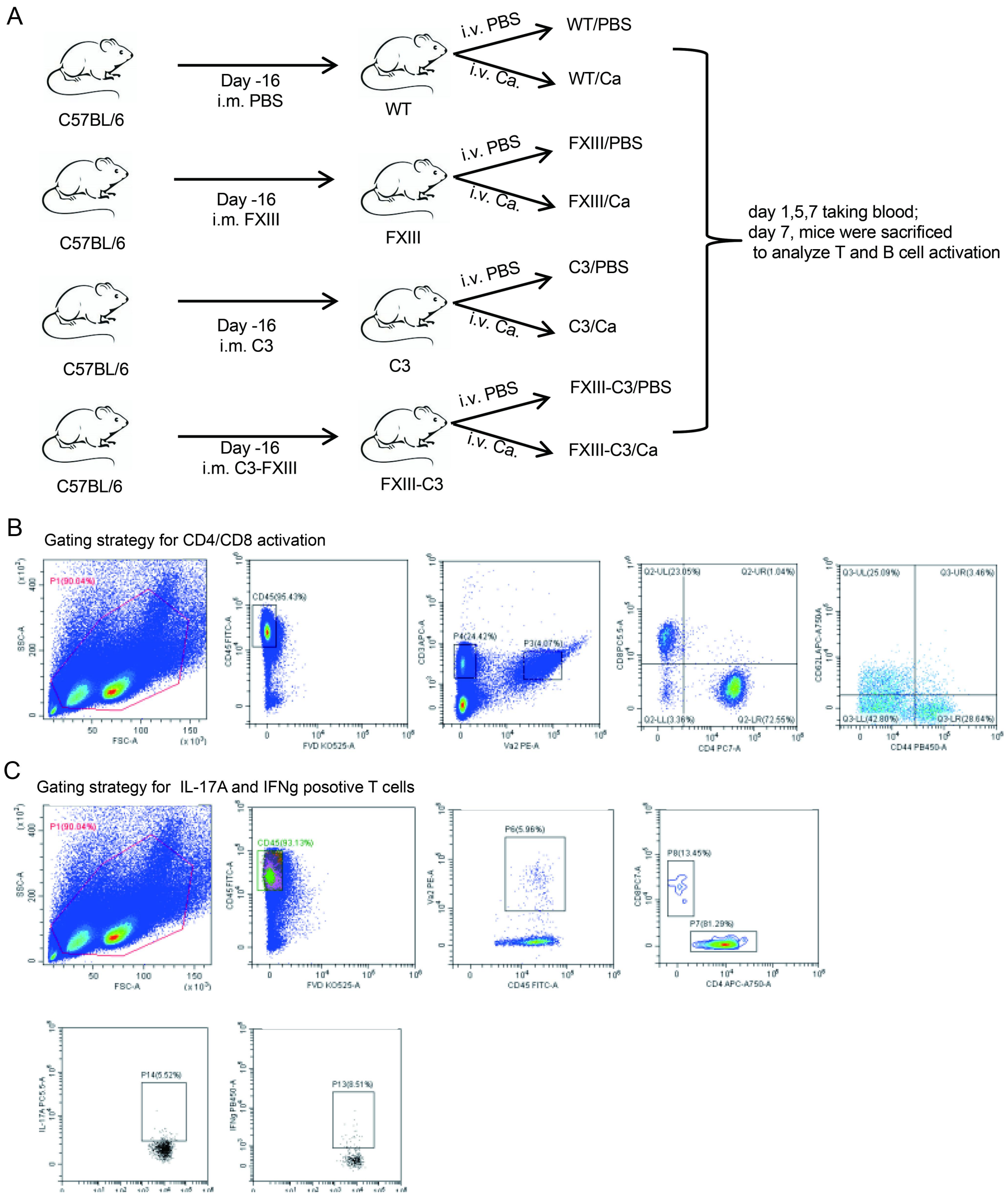

Supplemental Figure 5. Autoimmune FXIII deficient mice displayed defective complement and T cell activation. (A) Experimental schedule. (**B**) Gating strategy for activated CD4+ and CD8+ T cells, which is CD44^{high} CD62^{low} population among CD4+ and CD8+ T cells. (**C**) Gating strategy for IFN-g and IL-17 positive CD4+ and CD8+ T cells. In the in vivo infection model, WT, FXIII, C3 and FXIII-C3 groups of mice were intravenously injected with *C. albicans* (5x10^4/mouse) or PBS, eight

groups of mice were clarified as WT/Ca and WT/PBS, FXIII/Ca and FXIII/PBS, C3/Ca and C3/PBS, FXIII-C3/Ca and FXIII-C3/PBS (n=6 per group). Flow scatter plots are shown from one of three independent experiments.

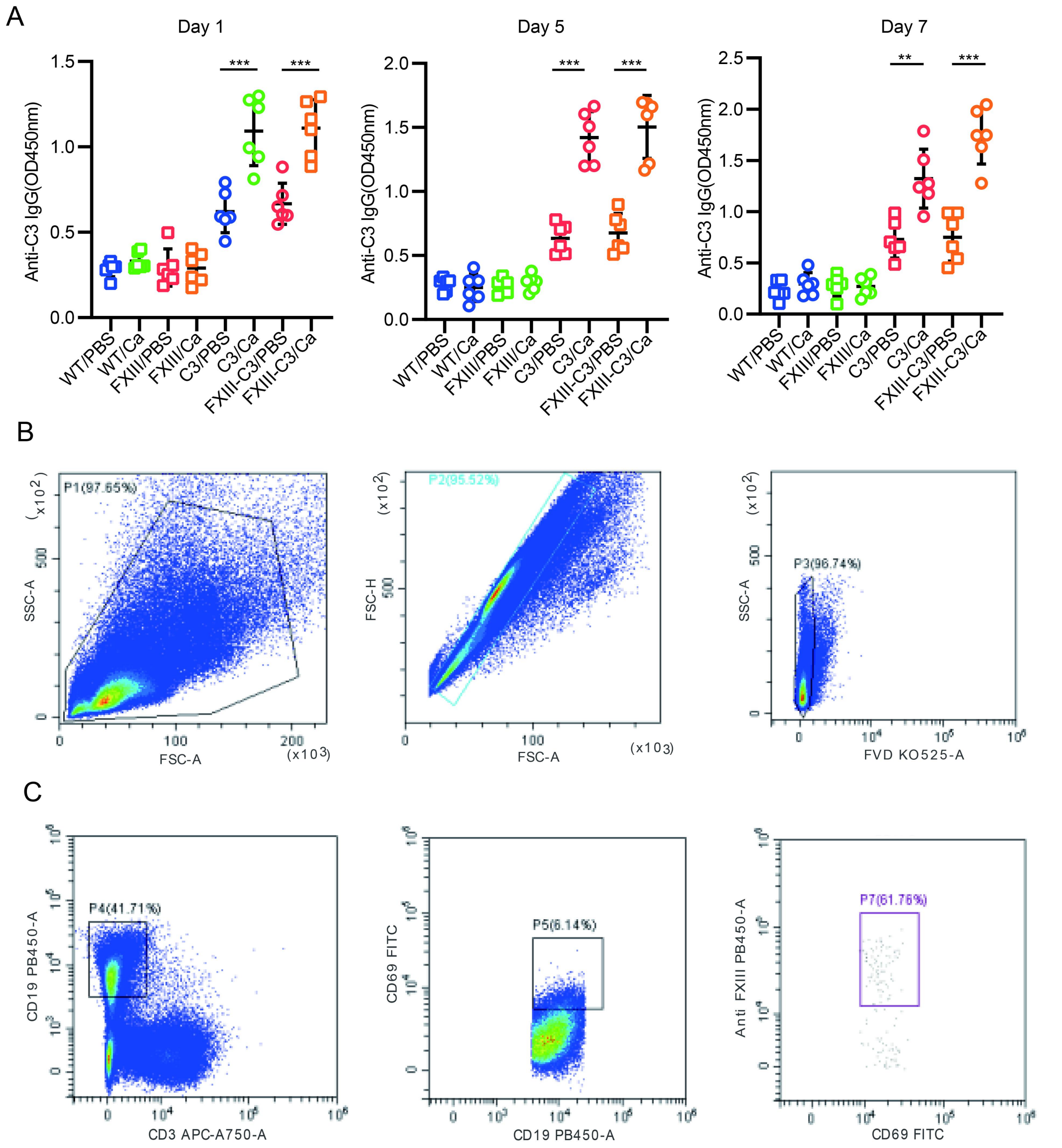

Supplemental Figure 6. Pathogenic *C. albicans* infections further induced auto-antibody generation in mice. (**A**) Levels of monospecific autoantibodies against C3 in eight groups of mice at day 1, 5, 7 post infection. C3 was coated, then different mouse plasma were added for further incubation. Then bound autoantibody was detected by anti-mouse IgG antibody. **(B and C)** Gating strategy for activated B cells (B) and auto-reactive subgroup of B cells (C), which are $CD19^{+}CD69^{+}$ B cell populations and anti-FXIII $IgG^{+}CD19^{+}CD69^{+}$B cell populations, respectively. In this

in vivo experimental settings, WT, FXIII, C3 and FXIII-C3 groups of mice were intravenously injected with *C. albicans* ($5x10^4$/mouse) or PBS, eight groups of mice were classified as WT/Ca and WT/PBS, FXIII/Ca and FXIII/PBS, C3/Ca and C3/PBS, FXIII-C3/Ca and FXIII-C3/PBS (n=6 per group). Data are shown as means ± SD. Unpaired two tailed Student's t-test was used for statistical analysis.*p<0.05, **p<0.01,***p<0.001.

Supplemental materials and methods

Measurement of FXIII antigen level and activity

FXIII-A antigen levels were quantified using a human FXIII antigen kit (HemoslL, 0020201300). Briefly, patient plasma was mixed and incubated with a latex bead solution coated with a specific anti-FXIII antibody. The turbidity of the solution was measured by recording the intensity of the transmitted light. FXIII-B level was measured using a sandwich ELISA with two pairs of anti-FXIII-B antibodies. Briefly, a monoclonal anti-FXIII-B antibody (1:200, SANTA CRUZ, sc-65957) was immobilized onto the wells overnight at 4 °C. After blocking and washing, diluted patient plasma or normal human plasma (NHP) was added and incubated for 2 h at 37 °C. Bound FXIII-B was then detected using a polyclonal rabbit anti-FXIII-B antibody (1:2000, Novus, NBP2-99778), followed by HRP-labeled goat anti-rabbit serum (Abcam, ab6721, 1:4000). The value for the Healthy control 2 (H2) sample was set to 100%, and the others were calculated accordingly. Human FXIII activity in all samples was quantified using the BIOPHEN™ Factor XIII kit (HYPHEN-Biomed, 227005) according to the manufacturer's instructions. Mouse FXIII-A level was analyzed using a mouse FXIII antigen kit (LSM Bio, ELI-02360).

ELISA

Purified combined autoantibody, isotype human IgG (Iso-IgG, BioLegend, 403502), or commercial anti-FXIII antibody (Proteintech, 66325-1-Ig) was coated overnight at 4 °C (1 μg/well). After washing, the wells were blocked with 4% milk powder, followed by incubation with C3 (CompTech, A113), C4 (CompTech, A105), or C5

(CompTech, A120) (1 μg/well in 100 μL PBS) for 1 h at 37 °C. Bound protein was detected using polyclonal goat anti-human C3 (CompTech, A213) , C4 (CompTech, A205), or C5 (CompTech, A220) antibodies and donkey anti-goat as a secondary antibody.

For detection of FXIII to C3, C3 was coated on the plates overnight at 4 °C (0.5μg/well). After washing, the wells were blocked with 4% milk powder, followed by incubation with different amount of FXIII in the presence or absence of thrombin and Ca^{2+} for 1 h at 37 °C. After washing, bound FXIII was detected using a polyclonal rabbit anti-human FXIII antibody (ABclonal, A1461, 1:2000) and HRP-labeled goat anti-rabbit serum (1:4000) as the secondary antibody.

Western blot and "Far Western" blot

To detect C3a release by Western blot (WB) analysis, the supernatant from the C3 cleavage assay (10 μL) was treated under reducing conditions, and samples were separated by SDS-PAGE using a 15% gel. Subsequently, the samples were transferred to a PVDF membrane. Following blocking with 5% milk powder, the membrane was incubated with a polyclonal rabbit anti-human C3a antibody (1:2000, CompTech, A218) and HRP-labeled goat anti-rabbit serum (1:4000) as the secondary antibody.

"Far Western" blot was applied to examine the specificity of purified anti-FXIII autoantibodies and autoantibodies secreted in the co-culture supernatant. Briefly, different amounts of purified anti-FXIII antibodies from P1 or co-culture supernatant were treated under reducing conditions, loaded onto a 12% gel, and separated by SDS-PAGE. Subsequently, the samples were transferred to a PVDF membrane.

Following blocking, FXIII was added as a ligand and incubated for 60 min at RT. After washing, bound FXIII was detected using a polyclonal rabbit anti-human FXIII antibody (ABclonal, A1461, 1:2000) and HRP-labeled goat anti-rabbit serum (1:4000) as the secondary antibody.

To analyzed whether C3 binds to denatured or linear FXIII, or FXIII binds to linear C3, again "far Western" was applied. Briefly, FXIII and C3 were treated under reducing conditions, then subjected to the SDS-PAGE, and transferred to PVDF membrane. Following blocking, the membranes were incubated with C3 or FXIII, then bound proteins were detected by polyclonal goat anti-human C3 (1:4000) or a polyclonal rabbit anti-human FXIII antibody (1:2000).

A phage display approach

C3 was first coated on the plates overnight at 4 °C (0.5μg/well). After washing, the wells were blocked with 4% milk powder, followed by incubation with pre purchased 7-phage-display-peptide-library(https://www.neb.cn/zh-cn/products/e8211-phd-7-phage-display-peptide-library-kit-v2) for 1 h at 37 °C. After extensive washing, bound bacteriophage were eluted by 0.2 M Glycine-HCl (pH 2.2). These eluted bacteriophage was then neutralized and infected *Escherichia coli* (ER2738) for amplification. These phages were further used for the second round screening. After three rounds screening, the eluted phages again infected ER2738 and plated on LB agar plates. 48 clones on the plates were randomly selected to analyze the binding ability to C3 using BSA as negative controls. Finally, two positive clones were selected, sequenced and aligned with FXIII sequence.
