# Supplementary material for: Combined FXIII-C3 autoantibodies elicit bleeding and complement dysfunction in autoimmune FXIII deficiency
Source: J Clin Invest. 2025 Dec 9;136(3):e192619. doi: 10.1172/JCI192619 (PMC12867163; doi:10.1172/JCI192619)

Figure 2C upper panel

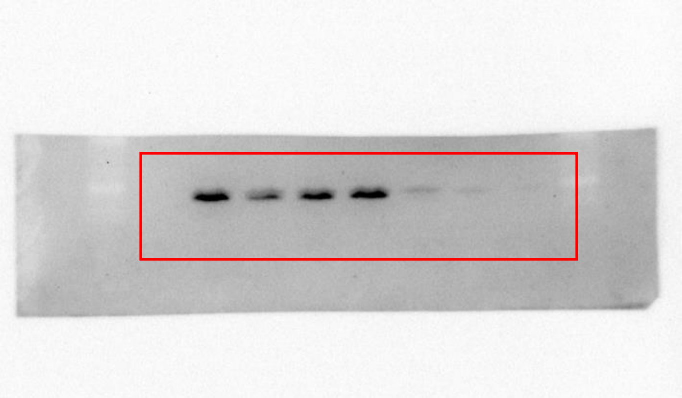

C3a

Figure 2C lower panel

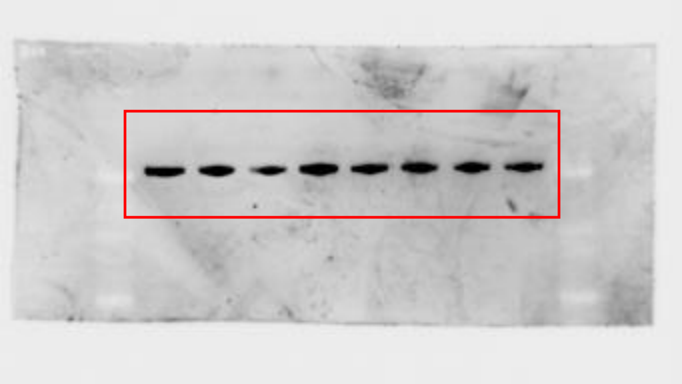

BSA

Figure 3A

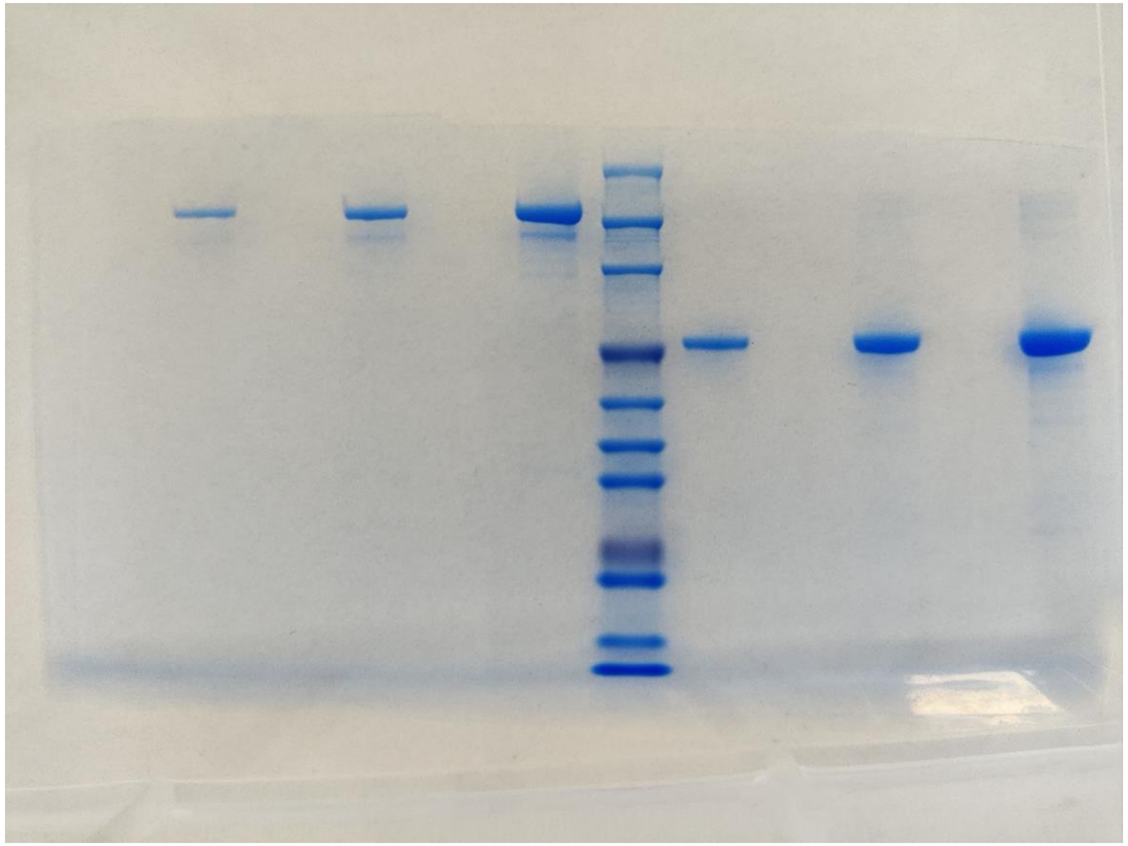

Figure 4 B

Upper two panels | Middle two panels | Lower two panels

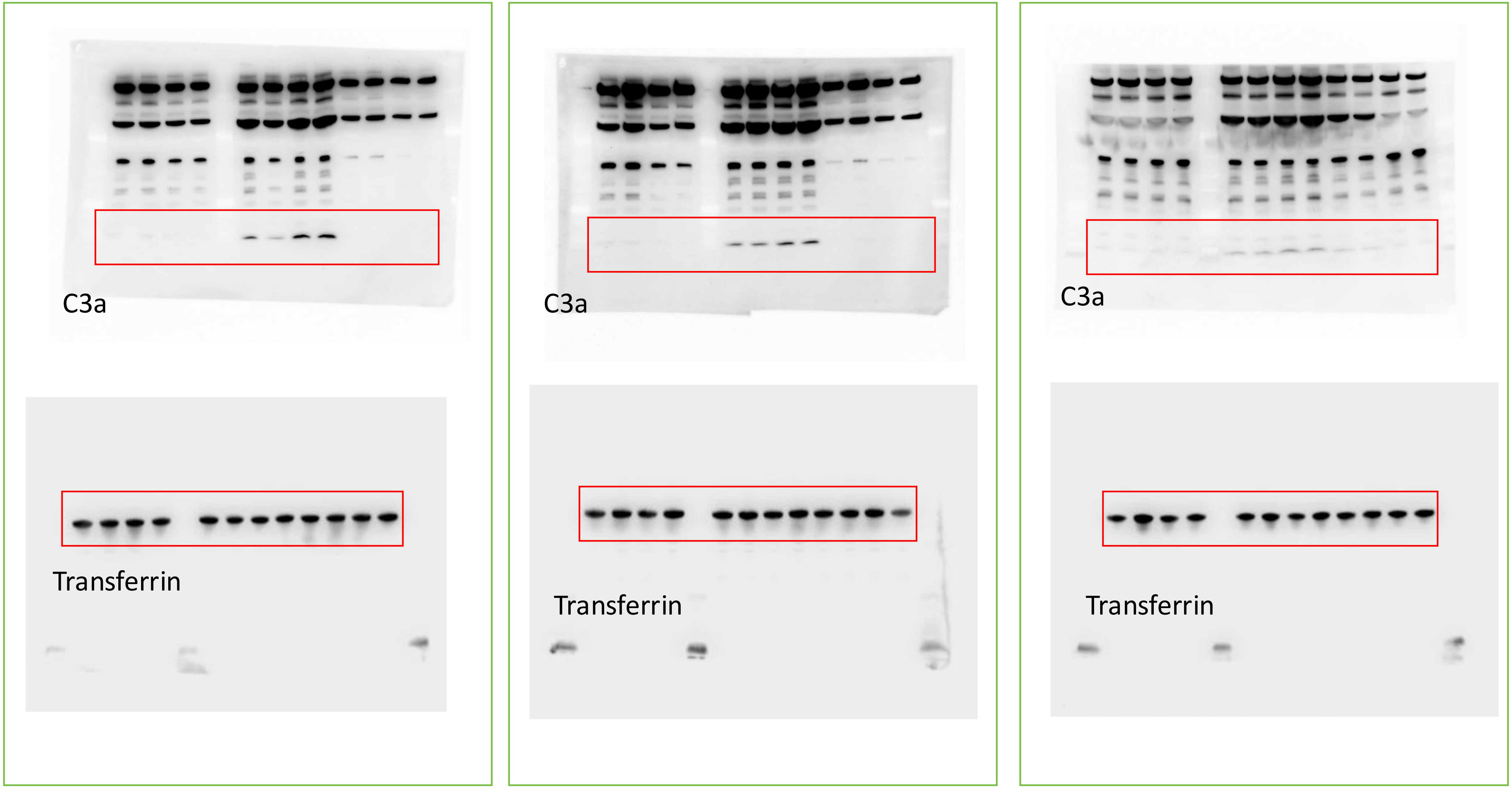

Figure 7E

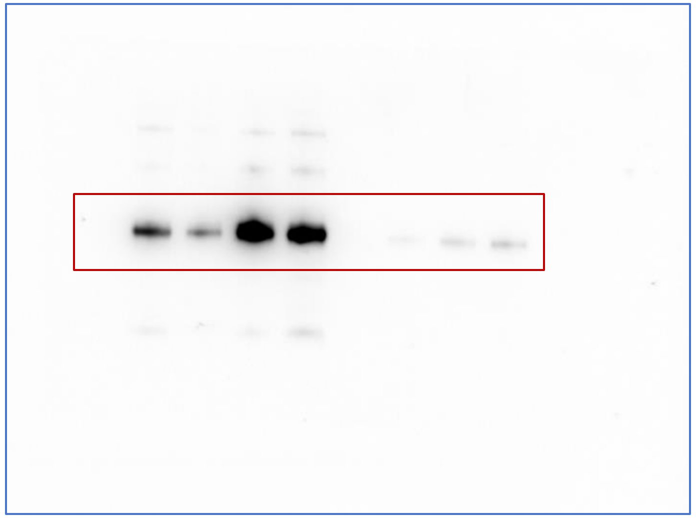

Line 1 2 3 4 5 6 7 8 9

Figure 7F

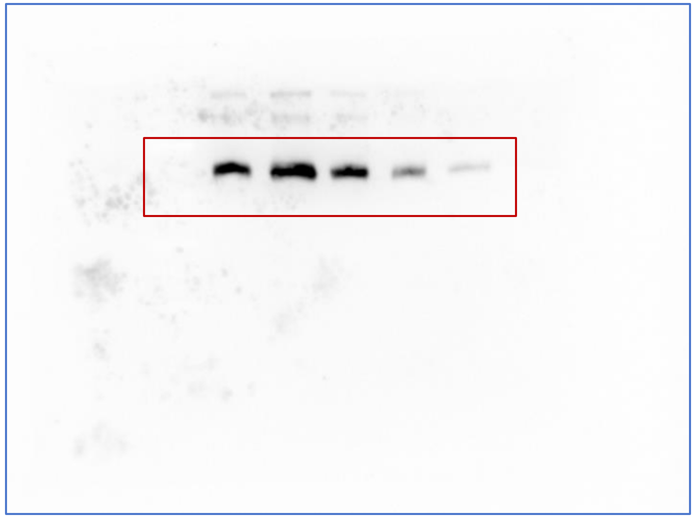

Line 1 2 3 4 5 6

Spplemental Figure 1E

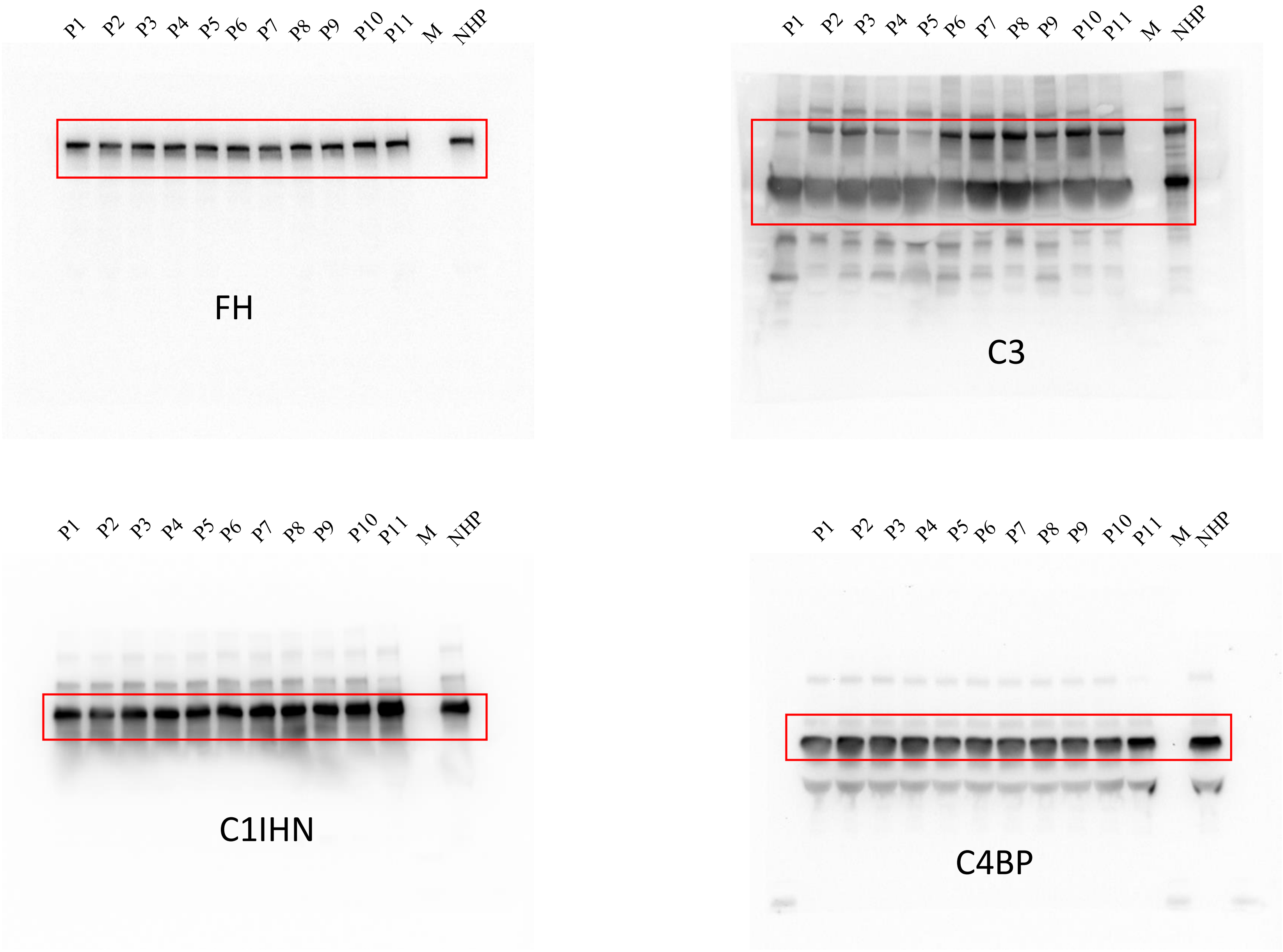

Spplemental Figure 1E

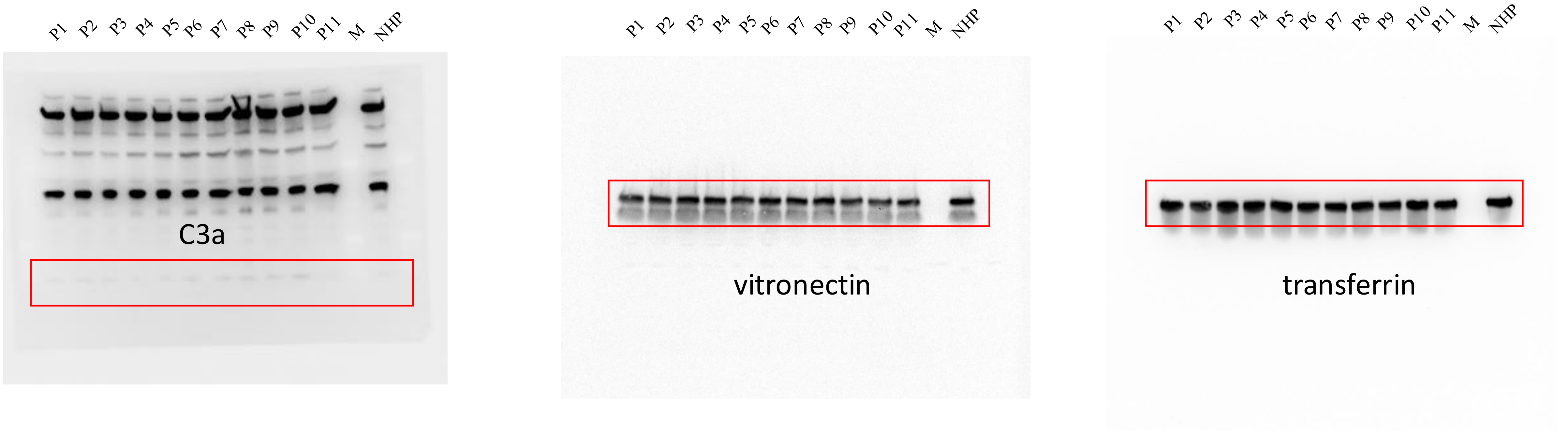

Supplemental-Figure 2A left panel

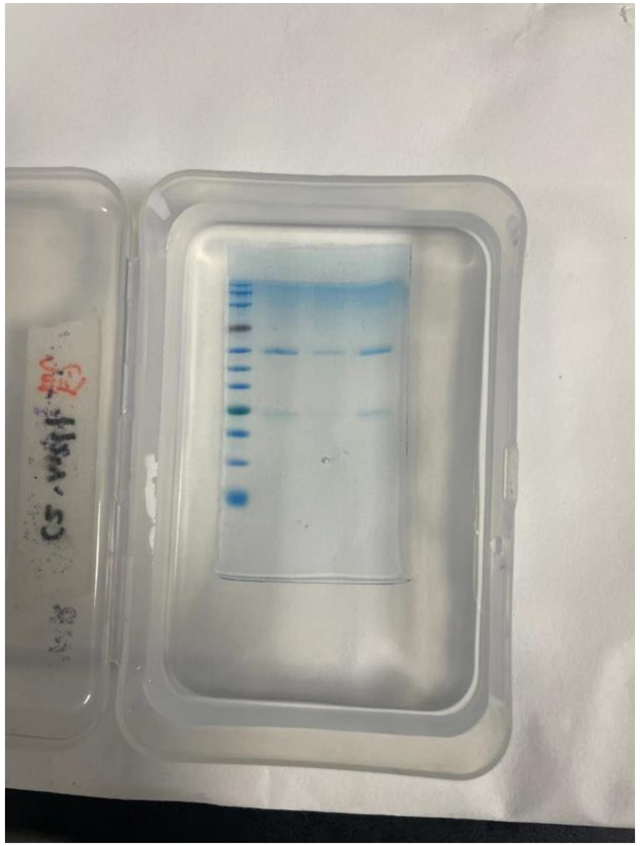

Supplemental-Figure 2A right panel

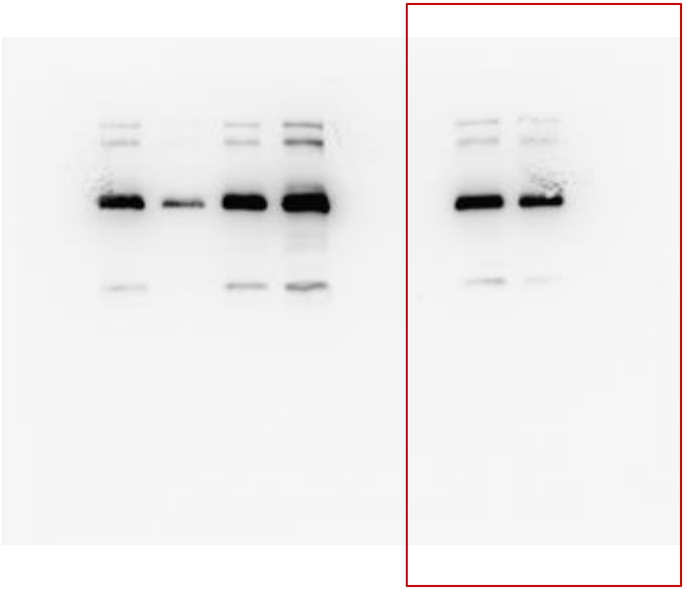

Supplemental-Figure 2B

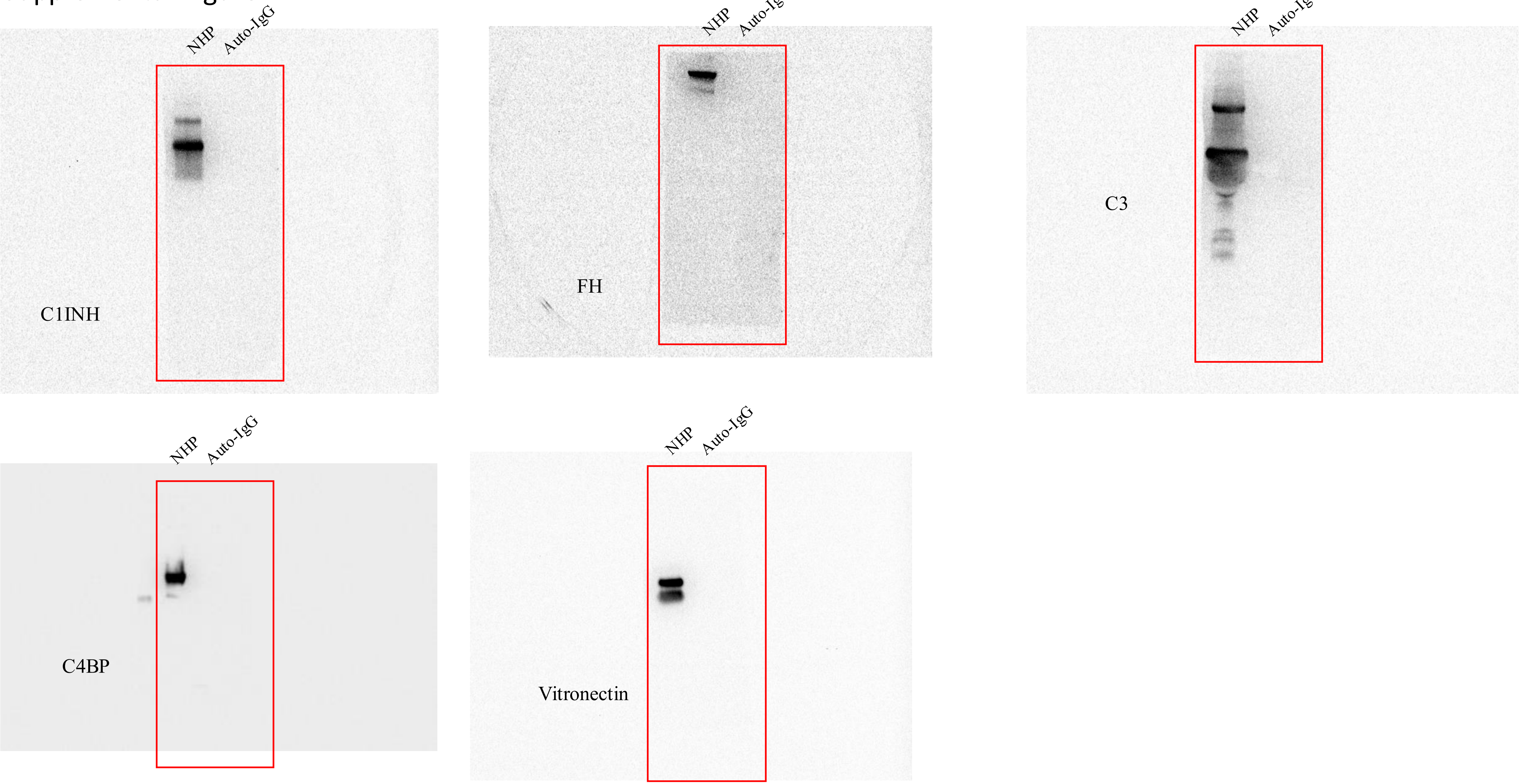

Supplemental-Figure 2C left

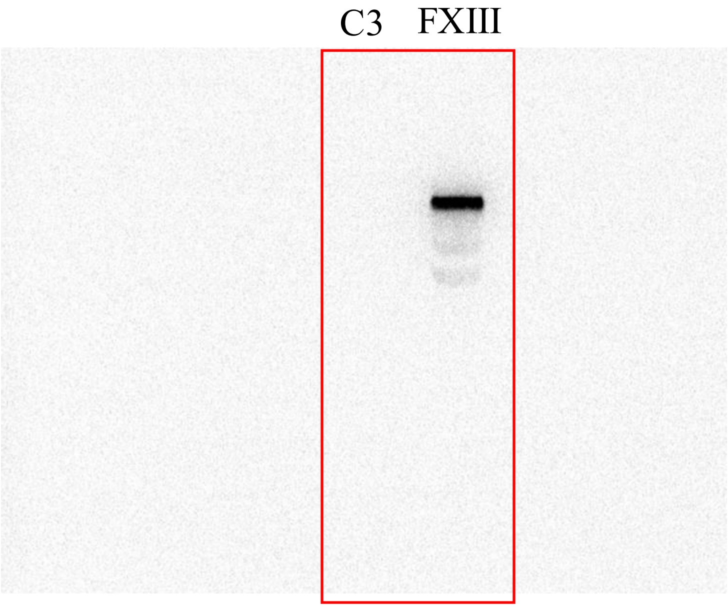

Supplemental-Figure 2C right

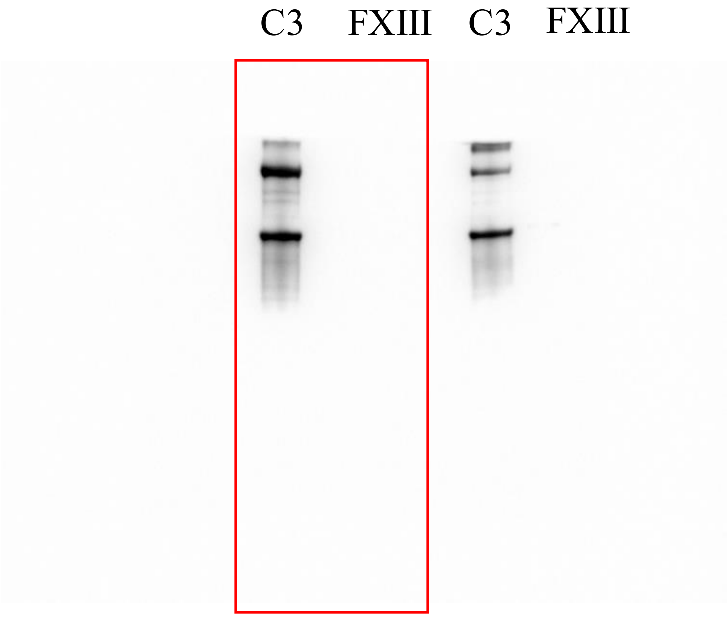

Supplement: Unedited blot and gel images [file jci-136-192619-s082.pdf]
